# Diphenylalanine Motif Drives Self‐Assembling in Hybrid PNA‐Peptide Conjugates

**DOI:** 10.1002/chem.202102481

**Published:** 2021-08-31

**Authors:** Carlo Diaferia, Concetta Avitabile, Marilisa Leone, Enrico Gallo, Michele Saviano, Antonella Accardo, Alessandra Romanelli

**Affiliations:** ^1^ Department of Pharmacy Research Centre on Bioactive Peptides (CIRPeB) University of Naples “Federico II” Via Mezzocannone 16 80134 Naples Italy; ^2^ Institute of Crystallography (CNR) Via Amendola 122 70126 Bari Italy; ^3^ Institute of Biostructures and Bioimaging (CNR) Via Mezzocannone 16 80134 Naples Italy; ^4^ IRCCS SDN Via E. Gianturco 113, 80143 Naples Italy; ^5^ Department of Pharmaceutical Sciences University of Milan Via Venezian 21 20133 Milan Italy

**Keywords:** assembly, beta sheet, fluorescence, peptide, peptide nucleic acid

## Abstract

Peptides and nucleic acids can self‐assemble to give supramolecular structures that find application in different fields, ranging from the delivery of drugs to the obtainment of materials endowed with optical properties. Forces that stabilize the “suprastructures” typically are hydrogen bonds or aromatic interactions; in case of nucleic acids, Watson‐Crick pairing drives self‐assembly while, in case of peptides, backbone hydrogen bonds and interactions between aromatic side chains trigger the formation of structures, such as nanotubes or ribbons. Molecules containing both aromatic peptides and nucleic acids could in principle exploit different forces to self‐assemble. In this work we meant to investigate the self‐assembly of mixed systems, with the aim to understand which forces play a major role and determine formation/structure of aggregates. We therefore synthesized conjugates of the peptide FF to the peptide nucleic acid dimer “gc” and characterized their aggregates by different spectroscopic techniques, including NMR, CD and fluorescence.

## Graphical abstract

**Forces triggering self‐assembly**. Conjugates of the PNA gc to the dipeptide FF assemble in water, producing fluorescent materials. Watson‐Crick hydrogen bonds between complementary nucleobases and aromatic interactions between amino acids could stabilize the aggregate. The latter mainly drive the aggregation process.

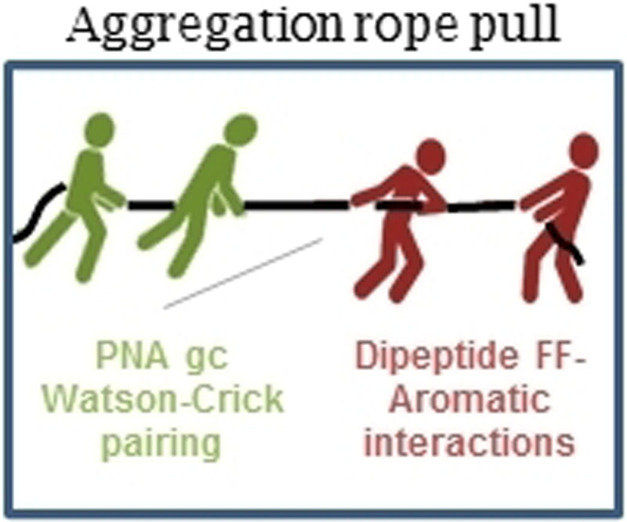

## Introduction

Self‐assembly of PNA‐based (PNA=peptide nucleic acid) molecules is the object of many recent investigations, due to the potential applications of these compounds in nanotechnology.[^1^, ^2^, ^3^, ^4^, ^5^, ^6^] PNA assemblies exhibit photoluminescence properties and morphologies that can be tuned by changing the base composition and covalently linking hydrophobic or aromatic moieties to the PNA chain to promote formation of supramolecular structures. Guanine‐containing sequences received particular attention; this base, in fact, can form three Watson‐Crick hydrogen bonds that are important for the stabilization of the aggregates. In addition, it is also known to produce complex structures such as quadruplexes.^[7]^ PNA guanine monomers (g), protected at the N terminus with the Fmoc (*N* ^α^‐9‐fluorenylmethoxycarbonyl) group and at the exocyclic amine with Bhoc (benzhydryloxycarbonyl), namely Fmoc−g(Bhoc)−OH, forms photonic crystals; conjugation of alkyl chains to Fmoc−g results in PNA amphiphiles that assemble in multiple morphologies, ranging from spheres to ribbons in different solvents.[^2^, ^8^] The amphiphiles form tetramers, as revealed by single‐crystal analysis, endowed with high mechanical stiffness and stability. A comparative study on all possible PNA dimers revealed that only guanine containing dimers produce ordered assemblies. More specifically, the PNA dimers of guanine‐cytosine (gc) generate aggregates that exhibit voltage‐dependent electroluminescence and fluorescence emission in a wide range of wavelengths.^[1]^ Moreover, the insertion of a Fmoc group at the N terminus of the dimer, to give Fmoc−gc, promotes assembly and yields aggregates characterized by very high quantum yields in organic solvents.^[9]^

We recently investigated the aggregation and the optical properties of hybrid peptide‐PNA compounds, in which the well‐known self‐assembling diphenylalanine (FF) peptide was covalently bound at the C terminus of PNA monomers and homodimers.^[10]^ Conjugates of FF to PNA dimers (*xx*‐FF, where x=adenine (a), thymine (t), g or c) aggregate at a concentration lower than conjugates to PNA monomers (a‐FF, g‐FF, c‐FF and t‐FF). The aggregation process of FF conjugates is mainly driven by stacking interactions between the phenyl rings; nevertheless, the role of the interactions mediated by nucleobases in determining the morphology of the aggregates is tangible. Indeed, PNA homodimer conjugates, independent of chemical nature of the base, form entwined fibers, while in case of conjugates to PNA monomers ordered structure are observed only with pyrimidines. Based on these observations, we found of a certain interest to extend our investigations to molecules in which the FF is conjugated to self‐complementary PNA heterodimers. Therefore, we designed novel hybrids coupling the FF peptide moiety to the “gc” PNA dimer. The gc may, in principle, stabilize aggregates by Watson‐Crick hydrogen bonds between complementary bases. We synthetized four different analogues H−FFgc−OH, H−gcFF−OH, H−FFgc−NH_2_ and H−gcFF−NH_2_ in which FF is alternatively positioned at the C or at the N terminus of the sequence and the C terminus is in its amidated or carboxylated form. The amidation of the C terminus allows to turn off one of the charges on the main peptide chain.

The aim of the present work is to investigate the aggregation of molecules composed by units, namely the peptide and the PNA that, taken separately, may aggregate thanks to different intermolecular forces, stacking interactions between phenyl rings and Watson‐Crick hydrogen bonds between complementary bases, respectively. In particular, we intended to explore whether the combination of peptide FF and the self‐complementary PNA sequence gc could result in a molecule able to exploit both intermolecular forces for the aggregation or only one of the two forces. In addition, we also studied the role of the composition of the C‐terminal end (carboxyl versus amide), the relative position of the peptide and of the PNA moieties on the structural organization and the fluorescence properties of the aggregates.

## Results and Discussion

### Synthesis and studies of the aggregation properties

**Figure 1:**
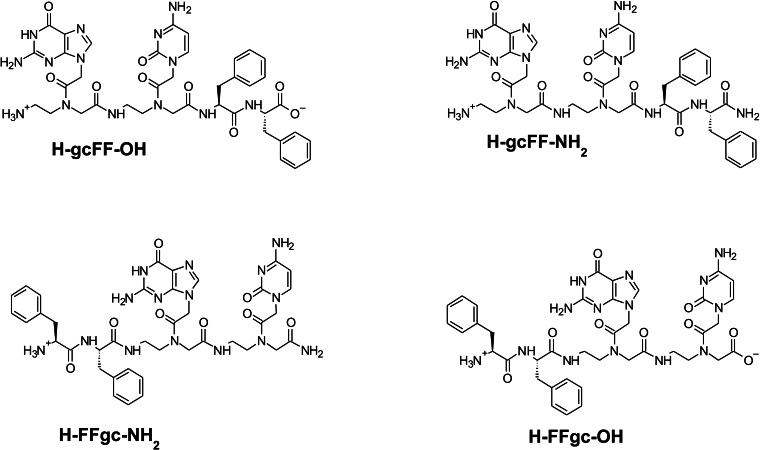
Schematic representation of FF‐PNA and PNA‐FF hybrid compounds.

All the hybrid peptide‐PNA derivatives, depicted in Figure 1, were synthesized according to solid‐phase peptide synthesis (SPPS), using Fmoc‐protected peptide and PNA monomers.[^11^, ^12^] Different resins were used as solid support to obtain the free carboxylic or amide group at the C terminus of the conjugates. After their purification by RP‐HPLC chromatography, all derivatives were chemically characterized by LC–MS and NMR spectroscopy (see Figures S1, S2, Supporting Information).

Irrespective of the presence of several aromatic groups, the derivatives show a good water solubility (up to 20 mg ⋅ mL^−1^) independently from the relative position of the FF dipeptide and the gc nucleobases. Analogously, the amidation of the C‐terminus does not affect significantly the solubility of the compounds in water. Initially, we assessed the aggregation ability of peptide derivatives by monitoring the formation of Phe‐excimeric species in solution by fluorescence measurements.^[13]^ Fluorescence spectra of PNA‐peptide conjugates (Figure S3) were recorded in water at several concentrations (0.005, 0.010, 0.050, 0.1, 0.5, 1.0, 5.0, 10.0 mg ⋅ mL^−1^), exciting the compound at 257 nm, which corresponds to the absorbance wavelength of the Phe residue (see Figure S4 for UV/Vis spectra of all the PNA‐peptide derivatives). Fluorescence spectra exhibit an emission peak around 310 nm, compatible with the emission of the excimers. The intensity of the peak shows two well‐distinguishable trends as a function of the concentration with an increase in the intensity of the peak for concentrations between 0.005 and 0.050 mg ⋅ mL^−1^, and a decrease for concentrations between 0.050 and 10.0 mg ⋅ mL^−1^. This behavior, previously observed for other aromatic‐based polypeptides such as F4 and F6, is symptomatic of the stacking between the phenyl rings and can be used for a qualitative evaluation of the range in which the aggregation occurs.[^14^, ^15^] In our case, experimental results indicate that all the hybrid derivatives start to self‐assemble in a concentration range between 0.050 and 0.10 mg ⋅ mL^−1^. A more accurate determination of the critical aggregate concentration (CAC) can be obtained by the fluorescence titration of the fluorescent dye, ammonium 8‐anilinonaphthalene‐1‐sulfonate (ANS), with increasing amount of each PNA‐FF derivative. Due to its spectroscopic features, ANS is a probe commonly used for the measurement of the CAC for supramolecular aggregates such as micelles, amyloid related aggregates and amphiphilic or aromatic peptides.[^16^, ^17^, ^18^] ANS is non‐emissive in water, whereas it emits fluorescence between 460–480 nm when is embedded in a hydrophobic compartment. CAC values are graphically extrapolated in the break point obtained plotting the fluorescence intensity of ANS in the maximum at 470 nm as function of the PNA‐peptide conjugate's concentration (see Figure S5). CAC values for each derivative are reported in Table 1. These values, in the micromolar range, are in good agreement with those found from the study of the excimer formation and they are comparable with CAC values previously measured for FF‐conjugated‐to‐PNA monomers and dimers (*x*‐FF and *xx*‐FF, where×=a, c, t, g).^[10]^

**Table 1:** Formula, theoretical molecular weight, retention time (R_t_), CAC value expressed in μm and mg ⋅ mL^−1^ of investigated conjugates.

Compound	Formula	R_t_ [min]	MW [Da]	CAC [μm]	CAC [mg ⋅ mL^−1^]
H−gcFF−OH	C_39_H_46_N_14_O_9_	9.82	854.3572	178	0.152
H−gcFF−NH_2_	C_39_H_48_N_15_O_8_	9.46	853.3731	170	0.145
H−FFgc−OH	C_39_H_46_N_14_O_9_	8.48	854.3572	78	0.066
H−FFgc−NH_2_	C_39_H_48_N_15_O_8_	8.34	853.3731	115	0.098

### Secondary structure aggregates characterization: FTIR, CD, CR and ThT assays

**Figure 2:**
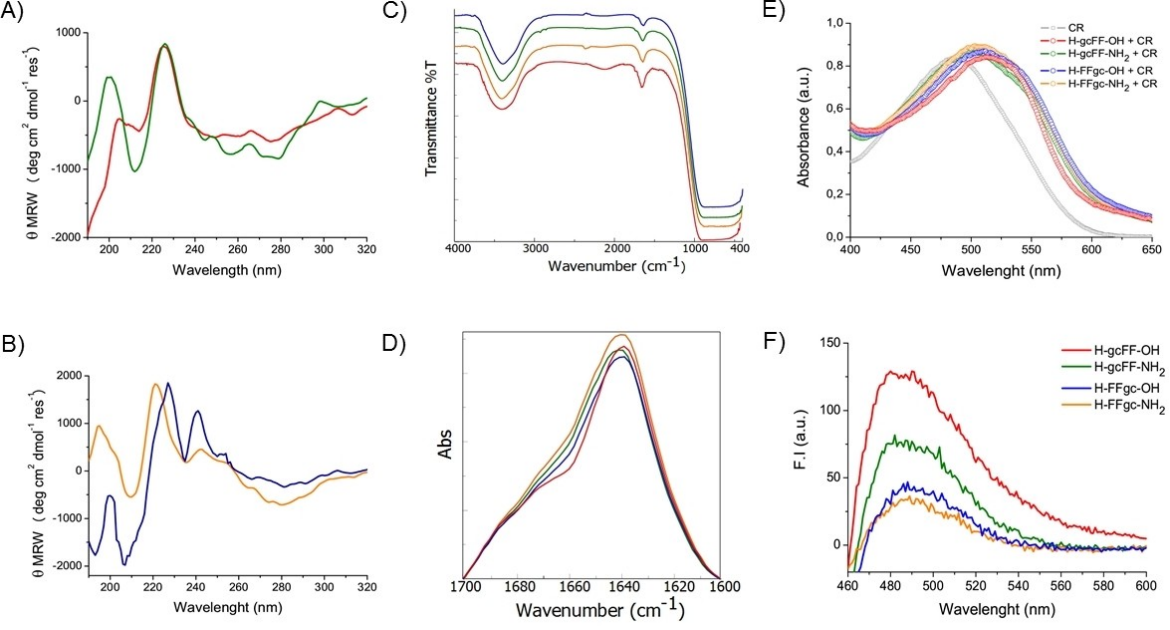
Secondary structure characterization. A) CD spectra of H−gcFF−OH (red) and H−gcFF−NH_2_ (green) at 5.0 mg ⋅ mL^−1^; B) CD spectra of H−FFgc−OH (blue) and H−FFgc−NH_2_ (orange) at 5.0 mg ⋅ mL^−1^. C) FTIR spectra of all PNA‐FF derivatives between 4000–400 cm^−1^. D) Absorbance deconvolution of the FTIR spectra in the amide I region (1700–1600 cm^−1^) shifted for a better visualization and comparison. E) UV/Vis spectra of PNA‐FF derivatives (2.5 mg ⋅ mL^−1^) co‐incubated with CR dye. E) Fluorescence spectra of ThT co‐incubated with each PNA‐peptide derivative after the subtraction of both PNA‐peptide and dye alone.

To gain insights into the supramolecular and global arrangement of the PNA‐FF conjugates, different spectroscopic techniques and specific assays were used: Circular dichroism (CD), Fourier‐transform infrared spectroscopy (FTIR), Congo red (CR) and Thioflavin T (ThT). Circular dichroism is an absorption spectroscopy technique sensible to elements of secondary structure.^[19]^ From the comparison of CD spectra, it is possible to point out analogies or differences in the supramolecular organization and to figure out the impact of the C terminus status. CD spectra of PNA‐FF conjugates, recorded between 320 and 190 nm in solution at 5.0 mg ⋅ mL^−1^, are reported in Figure 2.

Signals appearing in these spectra are the result of contributions deriving from amino acids and nucleobases. CD spectra of H−gcFF−OH and H−gcFF−NH_2_ (Figure 2A) exhibit an indicative and superimposable maximum at 226 nm, typical of the aggregative state of the diphenylalanine moiety.^[20]^ Generally, this specific dichroic signal is reported as minimum. The inversion of the dichroic signal could be attributed to an apparent inversion of the chiral configuration in the aggregates probably related to the intramolecular interaction network; this behavior was recorded by us for peptide‐based analogues systems, too.^[21]^ Alternatively, it is possible that the inversion of the signals is due to the twisting of the sheets.^[22]^ Indeed, it is reported for β‐sheet rich proteins that twisting of the sheets may result in a shift and also in the inversion of the CD signals. Beyond this peak, there is an additional relative maximum around 200 nm. This latter peak appears more intense for amidated derivatives than for carboxylated ones. This different intensity is probably due to the residual charge on the carboxylate derivative H−gcFF−OH, which exerts a repulsion effect and in turn disturbs the stacking between the PNA bases. Analogously to H−gcFF−OH and H−gcFF−NH_2_, also H−FFgc−OH and H−FFgc−NH_2_ have a CD spectrum dominated by a maximum (Figure 2B). This signal is located at 221 nm for H−FFgc−NH_2_ but it is red‐shifted at 228 nm for H−gcFF−OH aggregates. Signals appearing at wavelengths higher than 240 nm are typical of nucleobases; the low intensity of signals in this region let us hypothesize very weak interactions between bases. Mutual comparison of all CD spectral signatures indicate that FF moiety drives the self‐assembling in all cases, that is, either when it is at the N and at the C terminus of the PNA. Furthermore, the charge state of the C terminus does not significantly affect the aggregation properties too. Additionally, FTIR spectroscopy has been widely used to monitor elements of secondary structure in peptide‐based materials, including fibers and hydrogels.[^21^, ^23^] IR spectra of PNA‐FF‐based aggregates in water solution at a concentration of 5.0 mg ⋅ mL^−1^ are collected in Figures 2C and S6. An intense band in the amide A region, between 3000 cm^−1^ and 3700 cm^−1^, characterizes all the spectra. This signal is attributed to the exposure of the aggregates to water, generating both an asymmetric and a symmetric O−H and N−H stretching. Another broader signal with respect to the amide A is detectable in the amide I region (1600–1700 cm^−1^) and it is centered at 1638 cm^−1^. Deconvolution profiles in absorbance of the amide I spectral region (Figure 2B) show a prominent band centered for all the samples at 1642 cm^−1^, suggesting a β‐sheet secondary structure for all the PNA conjugates.^[24]^ An additional weak signal, more evident for H−gcFF−OH, can be detected at 1685 cm^−1^. This weak band is reported to be indicative for an antiparallel strand organization in the aggregates. However, this signal is wide, and could also be due to residual trifluoroacetic acid.^[25]^ These structural data support the experimental suggestion obtained by CD analysis that the PNA‐peptide conjugates form β‐sheet structures and that the charge on the C terminus does not affect the resulting supramolecular organization. The β‐sheet structuration was further confirmed by Congo red (CR) and Thioflavin T spectroscopic assays. CR is the sodium salt of 3,3′‐([1,1′‐biphenyl]‐4,4′‐diyl)bis(4‐aminonaphthalene‐1‐sulfonic acid) and represents a water‐soluble diazo dye used as a biological staining agent of amyloids deposits and for identification of β‐sheet‐rich materials.[^26^, ^27^] As a consequence of interaction with β‐aggregates, CR becomes torsionally restricted, thus inducing a substantial modification of the spectral imprint.^[28]^ Specifically, a characteristic increase of CR absorption is associated to a bathochromic effect from 480 to ∼540 nm. UV/Vis spectra of PNA‐FF aggregates (2.5 mg ⋅ mL^−1^) co‐incubated with CR, in comparison with CR alone, are reported in the range 400–650 nm (see Figure 2E). For all the samples, UV/Vis spectra show a bathochromic shift of the dye maximum, which further reinforces the hypothesis of a β‐sheet structuration. The organization of PNA‐FF conjugates in β‐sheet structures was also confirmed by the ThT assay. ThT is a fluorescent cationic benzothiazole dye that binds itself on the aggregates surface in a stabilized fashion.^[29]^ This phenomenon greatly enhances and shifts the fluorescence emission from 445 to 482 nm as a consequence of a loss of rotational freedom in the dye structure. Fluorescence spectra, reported in Figure 2F, show the emission peak obtained by subtracting spectra of each PNA‐FF derivative co‐incubated with 50 μm ThT and the peptide derivatives alone at the same concentration (20 mg ⋅ mL^−1^). The intensity of the emission peak, for all the PNA‐FF derivatives, does not change over time (up to 24 h). This result suggests that the aggregation kinetics are very fast and that no significant variations occur after the initial aggregation process. The relative intensity of the four samples are slightly different with a higher intensity for the carboxylated peptides than for the amidated ones. The major intensity of the emission in carboxylated peptide derivatives can be probably ascribed to favorable electrostatic interactions occurring between the positive charge of the ThT and the negative charge of the derivative.

### Scanning electron microscopy (SEM)

**Figure 3:**
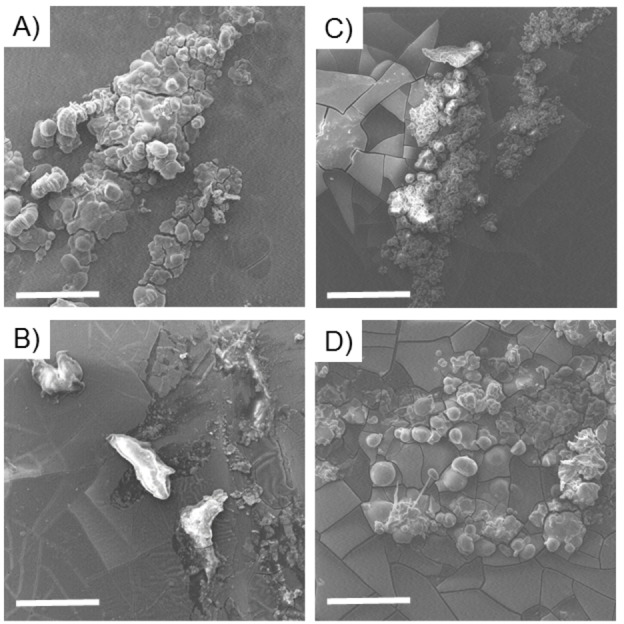
Selected SEM micrographs for self‐assembled hybrid PNA‐peptide systems drop‐cast from a 20 mg ⋅ mL^−1^ solution: H−gcFF−OH (A), H−gcFF−NH_2_ (B). H−FFgc−NH_2_ (C) and H−FFgc−OH (D). Magnification and scale bar for all the samples correspond to 2450× and 30 μm.

Information on the morphology of hybrid PNA‐peptides were obtained by the scanning electron microscopy (SEM) technique. Samples were drop‐cast on a stub from a 20 mg ⋅ mL^−1^ peptide solution and air‐dried at room temperature. From the inspection of micrographs reported in Figure 3, it can be observed that all the samples, regardless of their sequence and their charge termini, self‐assemble into spherical aggregates in the micro‐ and nanometer range. However, it seems that derivatives in which FF is at the N terminus exhibit a more regular shape. The formation of similar networks of nanospheres was previously observed for SEM images of Fmoc−gc, in which the gc PNA‐dimer is derivatized at the N terminus with the Fmoc protecting group. It can be supposed that the aromatic stacking between Phe residues simulates the interactions of the Fmoc groups.

### Fluorescence studies

**Figure 4:**
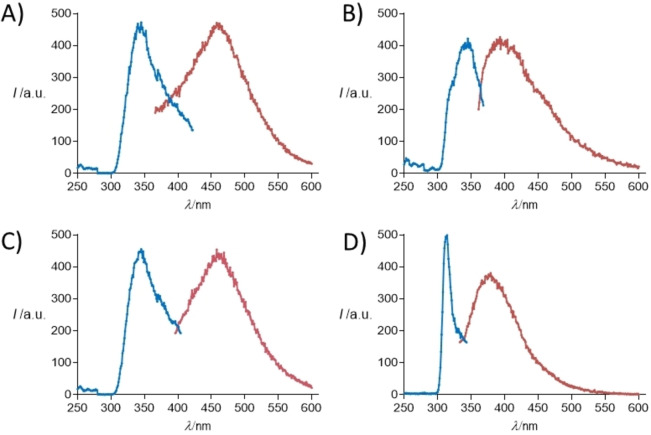
Excitation spectra in blue and emission spectra in red recorded in water for A: H−FFgc−NH_2_ (λ_ex_=340 nm, λ_em_=460 nm); B: H−gcFF−NH_2_ (λ_ex_=340 nm, λ_em_=400 nm); C: H−FFgc−OH (λ_ex_=340 nm, λ_em_=460 nm); D: H−gcFF−OH (λ_ex_=310 nm, λ_em_=380 nm).

**Figure 5:**
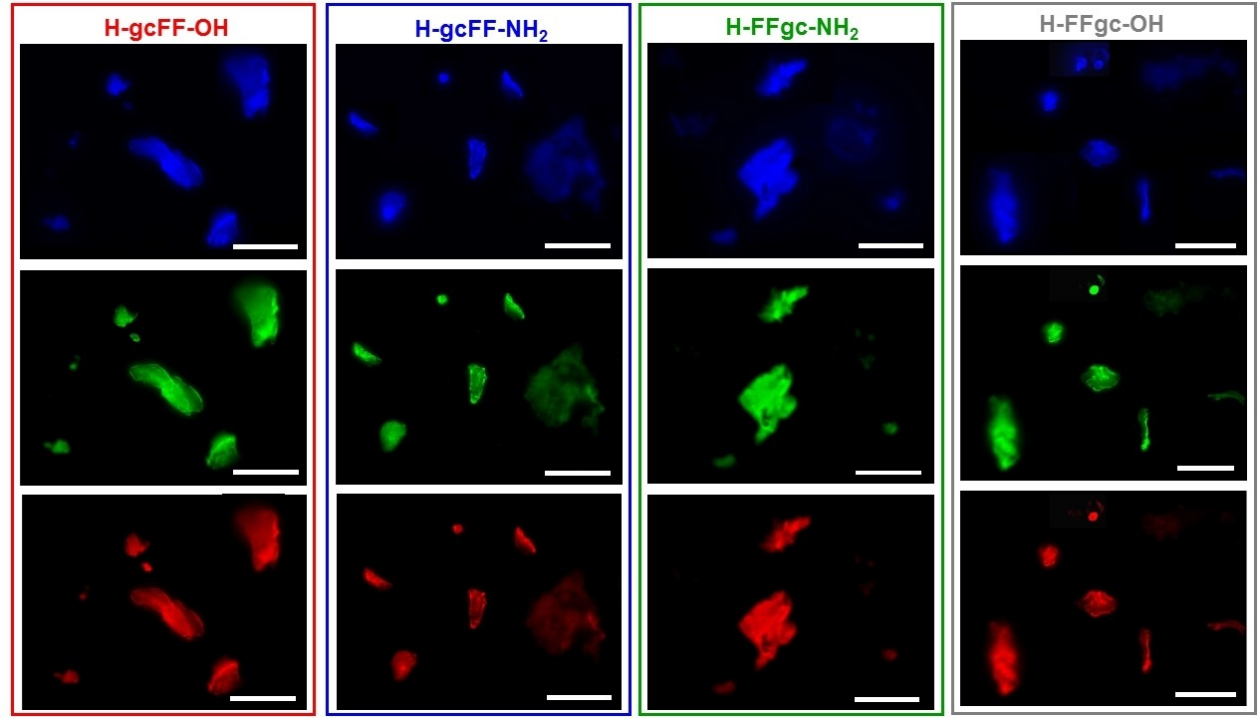
Fluorescence microscopy images of self‐assembled PNA‐FF derivatives drop‐cast on glass slides and air‐dried at room temperature. Samples are obtained by drop‐casting 10 μL of each PNA‐FF solution at a concentration of 20.0 mg ⋅ mL^−1^. Images are obtained by exciting samples in the spectral region of: DAPI (4’,6‐diamidino‐2‐phenylindole; λ_exc_=359 nm, λ_em_=461 nm); GFP (Green fluorescent protein; λ_exc_=488 nm, λ_em_=507 nm) and Rho (Rhodamine; λ_exc_=555 nm, λ_em_=580 nm). The scale bar of all the images corresponds to 50 μm.

Fluorescence studies were performed to determine optoelectronic properties of all the peptide‐PNA derivatives both in solution and at the solid state (Figures 4 and 5) and to investigate the role of nucleobases interactions in the stabilization of the aggregates (Supporting Information). Firstly, we investigated the optical behavior of conjugates in solution, prepared at a concentration of 20 mg ⋅ mL^−1^ in water in the range 300–430 nm. The excitation and the emission spectra for each sample are reported in Figure 4.

As expected on the basis of the literature, all hybrid conjugates exhibit fluorescence emission in the blue/green visible regions. In details, the two derivatives with PNAs at the N terminus show a maximum in the fluorescence emission around 400 nm; these maxima are consistent with those already reported for aggregates of nucleobases and for PNA homodimer‐FF aggregates. Instead, when the PNAs are at the C terminus, the maximum in the fluorescence emission shifts to 460 nm. This wavelength is typically observed for self‐assembled FF nanostructures assuming a β‐sheet conformation. As a consequence, the appearance of the signal at such a wavelength let us hypothesize the existence of a dense network of hydrogen bonds, similar to those observed in amyloid fibrils and in peptide nanostructures arranged into β‐sheets. On the other hand, it seems that the capping of the carboxyl group at the C terminus does not affect significantly the fluorescence properties of our compounds. Aggregated structures are able to keep their optoelectronic properties in the solid state as demonstrated by the fluorescence microscopy images reported in Figure 5 in the blue, green and red spectral regions. The robustness of peptide films against photodegradation and bleaching, which represents an essential feature to pursue any nanophotonic application, were also evaluated. Photodegradation was evaluated by comparing the fluorescence bleaching rate of the drop‐cast aggregated structures under continuous excitation for 4 h in the blue spectral region (DAPI, λ_exc_=359 nm).

In Figure S7A, selected immunofluorescence images recorded at different time points are reported. The images clearly show a gradual decrease of the fluorescence intensity upon time; with a decrease down to half of the initial fluorescence value after 45 minutes (Figure S7B). This time is much higher than that of conventional protein dyes.^[30]^

To investigate the role of the nucleobases’ interactions in the stabilization of the aggregate structures, we performed a fluorescence assay using the light‐up probe thiazole orange (TO) specific for the detection of duplexes. A similar approach was successfully applied to demonstrate duplexes mediated assembly in tetrakis(*p*‐hydroxyphenyl)methane conjugated to the DNA dimer gc.^[31]^ TO is an asymmetric cyanine, displaying weak fluorescence emission in aqueous solution and enhanced emission when bound to double stranded oligonucleotides due to restriction of the torsional motion between the benzothiazole and the quinoline moieties around the monomethine bond.^[32]^ The increase in the fluorescence emission around 530 nm is strongly dependent on the duplex nature and on the sequence; an over 50‐fold increase in the fluorescence emission is reported upon binding of TO with DNA duplexes, while a modest (3‐fold) increase is observed with PNA/DNA duplexes or with short PNA duplexes (Figure S8).^[33]^ Incubation of TO with H−FF−gc−NH_2_ at different ratios (ranging from 1 : 75 to 1 : 1 mol/mol TO/H−FFgc−NH_2_) did not result in any increase in the fluorescence intensity of the dye at 530 nm. The fluorescence spectra only show a band around 630 nm, which is typical of the aggregated form of the dye.^[34]^ These data highly suggest the absence of interactions between the nucleobases.

### NMR studies

Solution 1D [^1^H] and 2D [^1^H, ^1^H] NMR techniques were employed to further analyze the conformational properties of FFgc/gcFF derivatives. All compounds were analyzed at concentrations higher than the CAC in H_2_O. Studies on amyloid‐like systems clearly indicate that solution NMR allows to observe disaggregated and/or small oligomers but not high molecular weight oligomers and protofibrils species.[^35^, ^36^] Accordingly, NMR measurements gave information on the low molecular weight species potentially present in solution.

The 1D [^1^H] NMR spectrum of H−gcFF−OH shows a very crowded H_N_ and aromatic proton region (Figure S9A), indicating that multiple conformers are present in solution. The sharpness of NMR lines further indicates the presence in solution of disaggregated species or small aggregates. Deeper analysis with 2D [^1^H, ^1^H] spectroscopy confirms this evidence as at least six different phenylalanine spin systems can be recognized (Figure S9B) while, instead of a single cross‐peak corresponding to the H5‐H6 correlation in between cytosine aromatic protons, five cross‐peaks are evident (Figure S9C).

Most of the conformational variability that is observed can be reconducted to the reduced rotation around the tertiary amide in the PNA backbone and the possible *cis* and *trans* isomerization: due to the presence of two PNA bases (c and g), four rotamers are expected (i. e., *cis*‐*cis*, *trans*‐*trans*, *cis*‐*trans* and *trans*‐*cis*) for the PNA fragment gc.^[37]^ Additional conformers may arise due to the phenylalanine rotational state and/or to the co‐existence of monomeric completely disaggregated forms and small aggregates. The NOESY 300 spectrum (Figure S9D) shows only diagonal peaks, thus supporting the fast tumbling of a system with a relatively low molecular weight. The absence of an imino proton signal of guanine at low field in the H−gcFF−OH spectra let speculate that it is solvent‐exposed and thus not involved in gc canonical base pairing.

Similarly, the complexity and the absence of relevant line broadening in the 1D [^1^H] spectrum of H−FFgc−OH (Figure S10A) indicate, again, the coexistence of multiple conformations and small species in solution. Two phenylalanine spin systems are evident in the H_N_ – aliphatic protons correlation region of the TOCSY spectrum (Figure S10B) along with multiple gc conformations that are visible in the H_N_ backbone region between 7.8 and 8.2 ppm (Figure S10B). Analysis of the aromatic region (Figure S10C) indicates five H5−H6 correlations for the cytosine, further stressing the presence of different conformational families. The NOESY spectrum (Figure S10D) contains only a few positive NOEs, thus pointing out the flexibility of the system.

Next, the solution conformational features of H−gcFF−NH_2_ were investigated and again the 1D [^1^H] (Figure S11A) and 2D [^1^H, ^1^H] spectra (Figure S11B, C, D) pointed out the presence of small flexible species in solution. Similarly to H−gcFF−OH, the TOCSY spectrum contains multiple Phe spin systems (Figure S11B) along with several H5‐H6 cross‐peaks (Figure S11C) instead of the canonical one that is expected in a rigid system for the two aromatic protons of the cytosine. These observations point out the coexistence of multiple conformers in solution. The NOESY spectrum with only a few cross‐peaks (Figure S11D) further demonstrates the lack of a rigid well‐organized structure.

**Figure 6:**
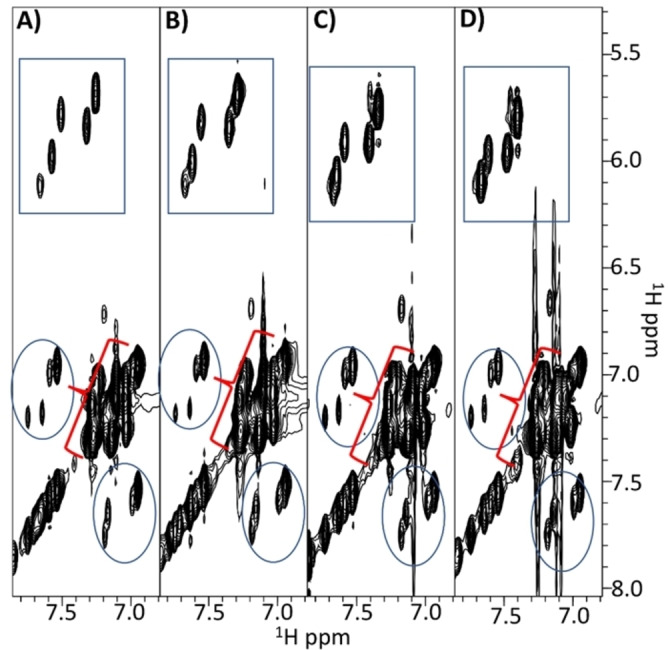
Comparison of 2D [^1^H, ^1^H] TOCSY spectra of H−FFgc−NH_2_ recorded in H_2_O/D_2_O 90/10 v/v at different concentrations (A: 1.3 mm, B: 2.6 mm, C: 14.2 mm, D: 46.9 mm). The region containing correlations from aromatic protons and the terminal CONH_2_ group are reported. Cross‐peaks arising from the H5‐H6 protons of cytosine are enclosed in rectangles, the cycles highlight cross‐peaks from the C‐terminal CONH_2_ group and the brackets point to cross‐peaks from aromatic protons in Phe residues.

Deeper studies were conducted with H−FFgc−NH_2_ for which spectra were recorded at increasing concentrations from 1.3 mm to 46.9 mm (Figure S12 and Figure 6).

1D [^1^H] (Figure S12) and 2D [^1^H, ^1^H] (Figure 6) spectra clearly indicate a dependence of the chemical shifts from the concentration. The pH value of the most concentrated sample (i. e. ∼50 mM) in H_2_O is 6.3 while 1–2 mm concentrated samples present pH values between 6.7 and 6.8.

**Figure 7:**
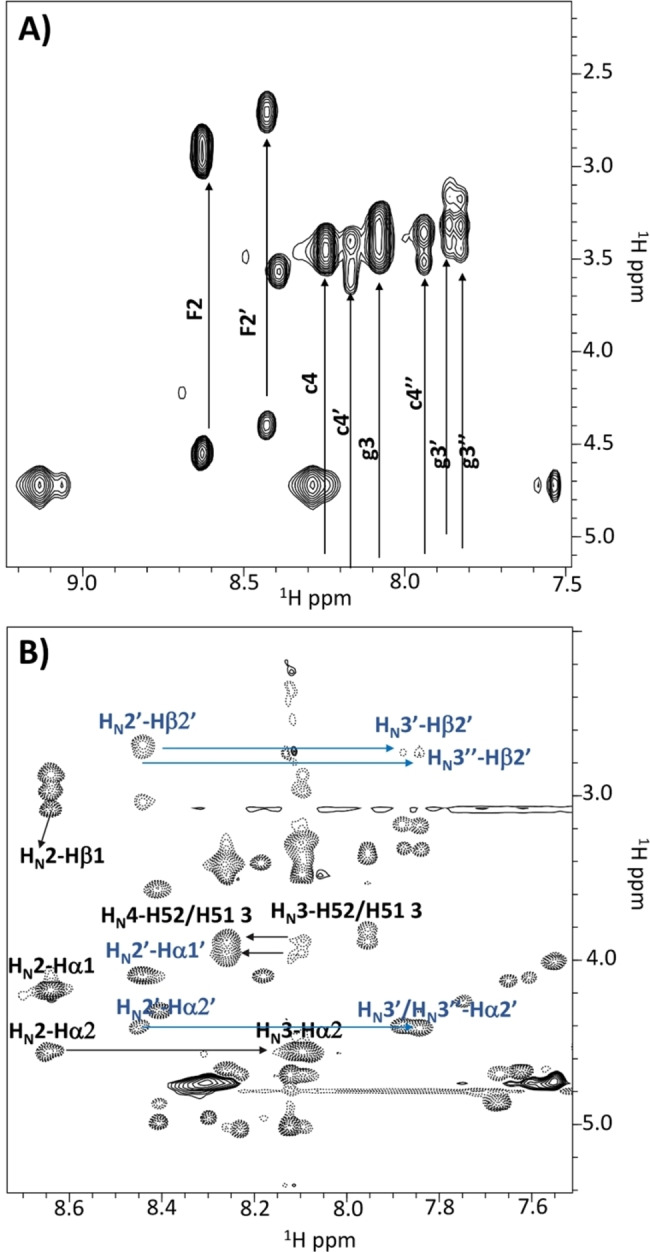
A) TOCSY and B) ROESY spectra of H−FFgc−NH_2_ at 46.9 mm concentration in H_2_O/D_2_O 90/10 v/v. Different spin systems are indicated in A) whereas a few sequential and intra‐residue ROE contacts are reported in B) where black and blue labels are used for residues belonging to different conformational families. As for the nomenclature, F1 and F2 stand for phenylalanine n.1 and n.2 using the one letter amino acid code and residue number; similarly, g3 and c4 stand for PNA‐guanine (=residue n.3) and PNA‐cytosine (=residue n.4); c4’, g3’ and c4”, g3” refer to other different rotamers.

The pH variation is not dramatic, thus it is possible that the observed chemical shifts variations are also pointing out aggregation phenomena rather than simply reflecting pH changes. In fact, the comparison of NOESY 300 spectra acquired at 1.3 and 46.9 mm concentrations (Figure S13) clearly evidences how the signs of NOEs start to change by raising the concentration due to formation of slower‐tumbling aggregated species. However, even at the highest explored concentrations (i. e., 14 and 46.9 mm), NMR spectra let observe in solution only low molecular weight species. The line broadening is reduced and multiple conformers are still evident even around 50 mm concentration as again witnessed by multiple cross‐peaks in the cytosine aromatic region of TOCSY spectra (Figure 6) and by the reduced number of cross‐peaks in the NOESY spectrum (Figure S13). Comparison of TOCSY and ROESY spectra allows partial resonance assignments for a few of the conformers in solution (Figure 7, Table S1) and reveals: the first amide H_N_ proton is fully exposed and in exchange with D_2_O; a prevalence of sequential strong H_N_i+1‐H_α_i signals canonical of random and/or extended β‐forms.

Nevertheless, as it can be seen in the 1D spectra (Figure S12) even at 46.9 mm concentration, the absence of a signal at low field arising from the imino proton let speculate that guanine and cytosine are not paired. It is worth noting that under the experimental conditions used to run these NMR spectra (i. e., pH between 6.8 and 6.3), the c and g bases should be largely present in solution in the non‐protonated forms, the p*K*_a_ for N3‐protonated cytosine being equal to 4.2 and 1.9–2.2 for the N7‐protonated guanine.[^38^, ^39^]

However, to get further insights into the structural organization of the aggregates, we performed a few NMR experiments under more controlled pH conditions in 50 mm phosphate buffer at pH 7.1. Even at the highest explored concentration (i. e., 25 mm) we could really observe mainly non‐exchangeable protons in the NMR spectra whereas all H_N_ are solvent‐exposed (Figure S14A); the 2D [^1^H, ^1^H] TOCSY spectrum (Figure S14B) still shows four rotamers in the H5−H6 cytosine correlation region; the 2D [^1^H, ^1^H] NOESY spectrum is canonical of a low molecular weight species (Figure S14C). All this is likely indicating the presence in solution of NMR‐invisible, very large and soluble aggregates and monomeric species that could instead be observed (Figure S14). Nevertheless, we could record a 1D [^1^H] spectrum in 50 mm sodium phosphate buffer at 80 μm concentration with a decent signal‐to‐noise ratio that is very similar to that recorded at 25 mm concentration, thus further supporting the concept that in buffer solution, even under experimental conditions that should favor Watson‐Crick base pairing, NMR allows to observe mainly monomeric disaggregated species.

In conclusion, the solution NMR analyses give information only on the disaggregated or small oligomeric species whereas the large aggregates characterizing these compounds are not visible due to the very fast relaxation. As the PNA skeleton is flexible, several rotamers are present in solution and an additional conformational variability is added by the two phenylalanines. Interestingly, when the phenylalanines are positioned at the C‐terminal side, they can sample a larger conformational space and give rise to a larger number of conformational families in solution as indicated by the greater number of spin systems that can be observed for them in contrast to the four spin systems (two for each F) characterizing the FFgc compounds. Moreover, no relevant differences in terms of structural features can be identified in the NMR spectra of H−FFgc−OH with respect to H−FFgc−NH_2_ or of H−gcFF−OH with respect to H−gcFF−NH_2_.

### Aggregates binding to DNA

With the aim to understand whether our molecules could be exploited to incorporate oligonucleotides to be applied as DNA/RNA sensors, we preliminarily investigated the ability of one selected aggregate, H−gcFF−OH, to interact with a single stranded DNA (Figure S15). The formation of the complex was monitored by comparing CD spectra of the DNA and of the selected aggregate with their mixture after annealing. The CD spectrum of the annealed mixture (Figure S15C) shows an intense positive band at 300 nm and a negative band at 278 nm; this band is very weak in the CD spectrum of H−gcFF−OH and it suggests the formation of a novel species in solution that can be identified as DNA‐aggregate adduct. The subtraction of the CD spectrum of the DNA from the CD spectrum of the annealed DNA+H−gcFF−OH mixture further confirms this hypothesis. Moreover, the CD spectrum of the annealed mixture does not change after 60 minutes from its preparation, thus suggesting that, in this period, the complex remains stable in solution. Thermal stability of the complex, monitored by CD, reveals that the complex is stable up to 49 °C. Although very preliminary, this result represents an indication towards future application of PNA‐peptide aggregates for the detection of oligonucleotides. These results, in agreement with the revealed structural data, let speculate a scenario in which intermolecular π‐π contacts between phenylalanines create a hydrophobic inner core that is masked from the aqueous polar environment while the PNA bases, being more polar and exhibiting a lower tendency to form intermolecular interactions, remain exposed at the edges of the aggregates available to participate in molecular recognition events.

## Conclusion

Investigations on aggregates formed by conjugates composed by the FF peptide and the gc PNA dimer furnish interesting results in term of forces that drive the self‐aggregation. Fluorescence properties suggest that in FFgc conjugates a dense network of hydrogen bonds is formed. Although CD spectra indicate formation of β‐sheets in all cases, NMR data clearly show that at least in solution there are many species in equilibrium. Interestingly, there are no indications that suggest interactions between nucleobases. These observations let us speculate on the strength of interactions that could stabilize our aggregates, where apparently contacts between aromatic amino acids responsible of β‐sheet formation seem to be more relevant than interactions between complementary nucleobases. The relative position of the peptide and the PNA affects aggregation and fluorescence properties of our molecules. When the PNA dimer is at the C‐terminal end of the molecule, aggregation occurs at a lower concentration as compared to molecules in which PNAs are at the N terminus, irrespective of the presence of a carboxyl or an amide at the C terminus. These findings open the way to new investigations aimed at discovering how to produce stable structures stabilized also by Watson‐Crick hydrogen bonds other than by π‐π stacking.

## Material and Methods

**Fluorescence microscopy and photostability study**: Hybrid PNA‐FF derivatives were dissolved in water at a concentration of 20.0 mg ⋅ mL^−1^. 10 μL of the resulting solutions were drop‐cast on a clean coverslip glass, dried and imaged with fluorescence microscopies. Immunofluorescence images were taken with an automated upright microscope system (Leica DM5500 B) coupled with Leica Cytovision software. The photostability of the self‐assembled aggregates structures was assessed on the same samples drop‐cast on glass. The sample was left under continuous excitation (spectral region DAPI, λ_exc_=359 nm) for 240 minutes and fluorescence images were recorded at several time points (each 15 minutes during the first 180 minutes and each 30 minutes during the remaining time). Fluorescence decay was reported as percentage respect to amount of the initial fluorescence.

**NMR characterization**: Solution NMR spectroscopy was employed to investigate the conformational properties of the following compounds: H−FFgc−NH_2_ (1.3, 2.6, 14.2 and 46.9 mm concentrations); H−gcFF−NH_2_ (1.73 mm concentration); H−FFgc−OH (1.52 mm); H−gcFF−OH (2.16 mm). Samples were prepared by dissolving compound powders in 540 μL of H_2_O/D_2_O 90/10 (v/v). NMR spectra were recorded at 298 K on a Varian Unity Inova 600 MHz spectrometer equipped with a cold probe.

For each sample, the 1D [^1^H] spectrum was recorded along with a set of 2D [^1^H, ^1^H] experiments. In detail, 2D [^1^H, ^1^H] TOCSY (Total correlation spectroscopy), NOESY (Nuclear Overhauser enhancement spectroscopy), ROESY (Rotating frame Overhauser enhancement spectroscopy) and DQFCOSY (Double quantum‐filtered correlated spectroscopy) spectra were recorded.[^40^, ^41^, ^42^, ^43^] 2D [^1^H, ^1^H] spectra were acquired with 16–64 scans, 128–256 FIDs in t1, 1024 or 2048 data points in t2. Mixing times for TOCSY, NOESY and ROESY experiments were 70, 300 and 250 ms, respectively. Water suppression was achieved by Excitation Sculpting.^[44]^ Proton resonance assignments were gained by the standard Wüthrich protocol.^[45]^ Chemical shifts were referenced to the residual water peak at 4.75 ppm. Spectra were processed with V NMRJ 1.1D (Varian, Italy) and analyzed with the software MestReNova^[46]^ and NEASY enclosed in the CARA software suite (http://www.nmr.ch/).^[47]^

## Conflict of interest

The authors declare no conflict of interest.

## Supporting information

As a service to our authors and readers, this journal provides supporting information supplied by the authors. Such materials are peer reviewed and may be re‐organized for online delivery, but are not copy‐edited or typeset. Technical support issues arising from supporting information (other than missing files) should be addressed to the authors.

Supporting Information
