# Supplementary material for: Diphenylalanine Motif Drives Self‐Assembling in Hybrid PNA‐Peptide Conjugates
Source: Chemistry. 2021 Aug 31;27(57):14307–16. doi: 10.1002/chem.202102481 (PMC8597081; doi:10.1002/chem.202102481)
Supplement: Supplementary file 1 — Supporting Information [file CHEM-27-14307-s001.pdf]

Chemistry–A European Journal

Supporting Information

Diphenylalanine Motif Drives Self-Assembling in Hybrid PNA-Peptide Conjugates

Carlo Diaferia[+], Concetta Avitabile[+], Marilisa Leone, Enrico Gallo, Michele Saviano, Antonella Accardo,* and Alessandra Romanelli*

Author Contributions

C.D. Investigation:Equal
C.A. Investigation:Equal
M.L. Data curation:Equal; Investigation:Equal
E.G. Investigation:Supporting
M.S. Resources:Equal
A.A. Conceptualization:Equal; Supervision:Equal; Writing – review & editing:Equal
A.R. Conceptualization:Lead; Supervision:Equal; Writing – review & editing:Equal

Materials and Methods

Fmoc-Phe-OH amino acid and the activating agent 2-(1H-7-Azabenzotriazol-1-yl)-1,1,3,3-tetramethyluronoium hexafluorphosphate (HATU) were purchased at IRIS Biotech GMBH. PNA monomers (Fmoc-G(Bhoc)-OH and Fmoc-C(Bhoc)-OH) were purchased at Link Technologies. Rink amide MBHA and Wang resins and the activators N-hydroxybenzotriazole (HOBT) and O-benzotriazole-N,N,N′,N′- tetramethyl-uronium-hexafluoro-phosphate (HBTU) were purchased at Novabiochem (Gibbstown, NJ, USA). Cl-trt-Resin was purchased from Sigma, Acetonitrile (ACN), dry N,N-dimethylformamide (DMF) and dichloromethane (DCM) was from Romil. All other reagents were from Sigma Aldrich (Merck).

Peptide-PNA conjugates synthesis

The dipeptide FF was synthesized following a standard Fmoc chemistry protocol using different resins, in order to obtain peptides containing a carboxylic acid or an amide at the C-terminus, respectively. [1]

H-gcFF-OH conjugate was obtained on a 50 μmol scale by solid phase peptide synthesis employing a Wang Chem-Matrix (0.39 mmol/g) with the following protocol: in a batch reactor, resin was covered with DMF and allowed to swell for 30 min. In a round-bottom flask 10.0 equivs. of the amino acid Fmoc-Phe-OH were dissolved in dry DCM under argon atmosphere. This solution was cooled to 0 °C and 5.0 equivs. of DIC were slowly added. The mixture was stirred for 20 min at the same temperature and DCM was evaporated under nitrogen. The residue was dissolved in DMF and added to the resin suspension with 0.1 equivs. of DMAP. The suspension was shaken at room temperature for 1 h. Resin was then capped, filtered, washed with DMF (3x), DCM (3x), MeOH (3x) and dried. The second amino acid and the PNA monomers were then anchored employing standard protocols for the solid phase synthesis.[2] H-FFgc-OH conjugate was synthesized on trityl chloride resin (1.09mmol/g) with the following protocol: after swelling of the resin beads in DCM (1 mL), the first PNA residue (0.06 mmol), dissolved in DMF, was attached to the resin in the presence of DIPEA for 1h. The other residues were attached on the resin employing standard protocols for the solid phase synthesis. Amidate conjugates (H-FFgc-NH_2 and H-gcFF-NH_2) were obtained employing standard protocols for the solid phase synthesis.·

To improve the coupling efficiency double couplings were carried out on PNA-guanine monomer. Purification of crude products was carried out by RP-HPLC on a Shimadzu LC-8A, equipped with a SPD-M10 AV diode array detector using a Phenomenex Synergy 4μm Fusion-RP (80 Å, 50 × 21.2 mm) column with a flow rate of 20 mL·min^{-1} and with a gradient of CH_3CN (0.1% TFA) in H_2O (0.1% TFA) from 5 to 50 % in 20 minutes. Products were lyophilized three times, the first to remove

the HPLC solvents, the second upon dissolution of the samples in H_2O/CH_3COOH 7/3 v/v, the third following dissolution in H_2O. After lyophilization, peptides were obtained with a purity of >95%. Pure compounds were analyzed by LC-MS on a LC-MS Agilent Technologies 6230 ESI-TOF on a Phenomenex Jupiter 3µ C18 (150 × 2.0 mm) column with a flow rate of 0.2 mL·min^{-1}. Formula, retention time (R_t), theoretical molecular weight of investigated conjugates are collected in Table 1.

Preparation of peptide solutions

Solutions of PNA-peptide conjugates were prepared by direct dissolution of powders in water. After proper dilutions, concentrations were analytically determined by UV-Vis spectroscopy on UV-Vis Thermo Fisher Scientific Inc (Wilmington, Delaware USA) Nanodrop 2000c spectrophotometer, equipped with a 1.0 cm quartz cuvette (Hellma), using the molar absorptivity ε_{260} of 18300 M^{-1} cm^{-1} for all the compounds.

Critical aggregate concentration (CAC) measurements

CAC values for PNA-FF and FF-PNA derivatives were obtained by fluorescence measurements, titrating the fluorescent probe ANS (8-anilino-1-naphthalene sulfonic acid ammonium salt) with each conjugates.[3,4] Fluorescence spectra were recorded at room temperature on a Jasco Model FP-750 spectrofluorophotometer in a 10.0 mm path length quartz cell. Small aliquots of compounds were added to 0.2 mL of aqueous solution of 200 µM ANS. The excitation wavelength was set at 350 nm, equal excitation and emission bandwidths (5 nm) and a 700 V Voltage were used throughout the experiment. Final spectra, to be used for calculations, were obtained after blank correction and adjustment for dilution. The ANS fluorescence intensity at 470 nm was followed as a function of the concentration of the titration agent. The CAC values were determined in the breaking point of the linear least-squares fitting of the fluorescence emission versus the concentration and were reported in Table 1. All experiments were carried out in duplicate.

Fluorescence Measurements

Fluorescence measurements on peptide-PNA derivatives at a concentration of 20 mg·mL^{-1} were performed on a VARIAN Cary Eclipse Fluorimeter. Emission spectra were recorded upon excitation at various wavelength in the range 310-490 nm; excitation and emission slits were set respectively at 5 and 10 nm for measurements and a voltage of 800 V was employed for the experiments. The emission spectra of excimers were recorded on samples at different concentrations of compounds (0.005, 0.010, 0.050, 0.1, 0.5, 1.0, 5.0, 10.0 mg·mL^{-1}). For this set of experiments, the excitation wavelength was set at 257 nm, excitation and emission slits were set respectively at 5 and 10 nm for

measurements and a 800V Voltage was used throughout the experiment. Final spectra, to be used for calculations, were obtained after blank correction.

Thiazole Orange (TO) fluorescence assay

A solution of the hybrid H-FFgc-NH_2 (200µL, 11.150 mM, in water) was employed for the experiments. After annealing (90° C 5 min, 4°C over night), fluorescence measurements were performed, adding : a) 0.148mM of TO to the hybrid (TO/ H-FFgc-NH_2 ratio was 1:75 mol:mol); b) 11.150 mM of TO to the hybrid solution (TO/ H-FFgc-NH_2 ratio was 1:1 mol:mol); for this set of experiments, the excitation wavelength was set at 468 nm, excitation and emission slits were set respectively at 5 and 10 nm and a 800V voltage was used throughout the experiment. Final spectra, to be used for calculations, were obtained after blank correction. In order to compare fluorescence of our samples to that of a PNA duplex, in which base stacking and pairing occur, TO fluorescence assays were carried out employing an 8 bp PNA duplex, named ds-PNA as a control formed after annealing of PNA 1 (sequence: gtgacggt) and PNA 1comp (sequence:accgtcac) and a TO/dsPNA1 ratios of 1:1. Further experiments were carried out incubating TO with single strands.

CD spectroscopy

Far-UV CD spectra of the PNA-FF derivatives in aqueous solution were collected on a Jasco J-810 spectropolarimeter equipped with a NesLab RTE111 thermal controller unit using a 0.1 mm quartz cell at 25 °C. Spectra were recorded from 320 to 195 nm using samples at a concentration of 20.0 $mg \cdot mL^{-1}$. Other experimental settings are: scan speed, 10 $nm \cdot min^{-1}$; sensitivity, 50 mdeg; time constant, 16 s; bandwidth, 1 nm. Each spectrum was obtained by averaging three scans and correcting for the blank contribute. Each sample was recorded in triplicate and spectra in Optical Density (O.D./mdeg) were obtained by averaging three scans.

CD binding experiments between a 55 bases single strand DNA and H-gcFF-COOH (1/10, mol/mol) were recorded at 25 °C in the 340-200 nm range and are the results of 3 scans. The DNA sequence is: ACC CGG CAG TGC CTC CAG GCG CAG GGC AGC CCC TGC CCA CCG CAC ACT GCG CTG C. H-gcFF-COOH was dissolved in water at a concentration of 2 $mg \cdot mL^{-1}$ and the complex was previously annealed by warming up at 95 °C and slowly cooling to 4 °C. Thermic denaturation experiments were carried out at a 1 °C/min scan speed, recording at 250 nm for the ssDNA and 280 nm for ssDNA/H-gcFF-COOH complex.

Fourier Transform Infrared spectroscopy (FTIR)

FTIR spectra of nucleobases and PNA-FF solutions at 5.0 mg·mL^{-1} were collected on a Jasco FT/IR 4100 spectrometer (Easton, MD) in an attenuated total reflection (ATR) mode and using a Ge single-crystal at a resolution of 4 cm^{-1}. For secondary structure estimation, all the spectral data were processed using a built-in software and reported in absorbance. Spectra were collected in transmission mode and then converted in emission. Each sample was recorded with a total of 120 scans with a rate of 2 mm·s^{-1} against a KBr background.

Congo Red spectroscopic assay: Congo Red (CR) spectroscopic assay was carried out by UV-Vis measurements on Nanodrop 2000c spectrophotometer. A stock solution of CR (3.5 mg in 500 μL) was freshly prepared in 10 mM phosphate buffer, at pH 7.4 and filtered through 0.2 μm syringe immediately prior to use. A small aliquot (5 μL) of this solution was diluted with water at 12.5 μM final concentration and the UV-Vis spectrum was recorded between 400 and 700 nm at room temperature. 60 μL of each PNA-peptide solution (20 mg·mL^{-1}) were added to CR solution to obtain a final peptide concentration of 2.5 mg·mL^{-1}. The sample was left in incubation for 15 min at room temperature, then absorbance spectra were recorded and background subtracted using a Congo Red spectrum in phosphate buffer as reference solution.

Thioflavin T (ThT) spectroscopic assay

Aggregation behavior of PNA-FF conjugates was assessed by using Thioflavin T (ThT). Thioflavin T associates rapidly with β-aggregated peptides giving rise to an enhanced emission at 482 nm. Fluorescence spectrum of an aqueous solution of ThT (50 μM) before and after the addition of peptide derivatives (20.0 mg·mL^{-1}) was recorded at 25°C after the peptide addition into the cuvette. Samples were excited at 444 nm and fluorescence emission spectra were recorded between 454 and 600 nm.

Scanning electron microscopy (SEM)

Morphological analysis of nanostructures was carried out by field emission scanning electron microscope (Phenom_XL, Alfatest). 10 μL of PNA-FF solution (at 20 mg·mL^{-1}) were drop-casted on an aluminium stub and air-dried. A thin coat of gold and palladium was sputtered at a current of 25 mA for 75 sec. The sputter coated samples were then introduced into the specimen chamber and the images were acquired at an accelerating voltage of 10 kV, spot 3, through the Secondary Electron Detector (SED).

NMR experiments in phosphate buffer, pH 7.1

Additional NMR spectra were recorded on a Varian Unity INOVA 500 MHz spectrometer for samples of H-FFgc-NH_2 in 50 mM sodium phosphate buffer at pH 7.1 (total volume 540 µL including 40 µL of D_2O) at 25 mM concentration, 2.1 mM concentration and 80 µM concentration.

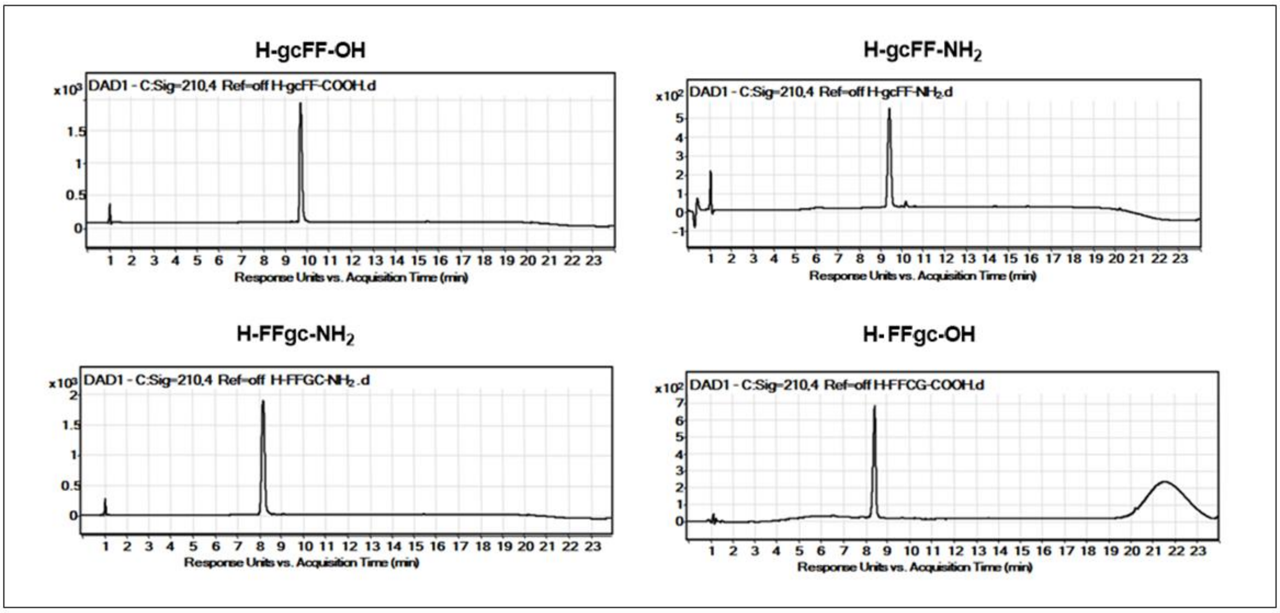

Figure S1: RP-HPLC chromatograms for hybrid PNA-peptide derivatives

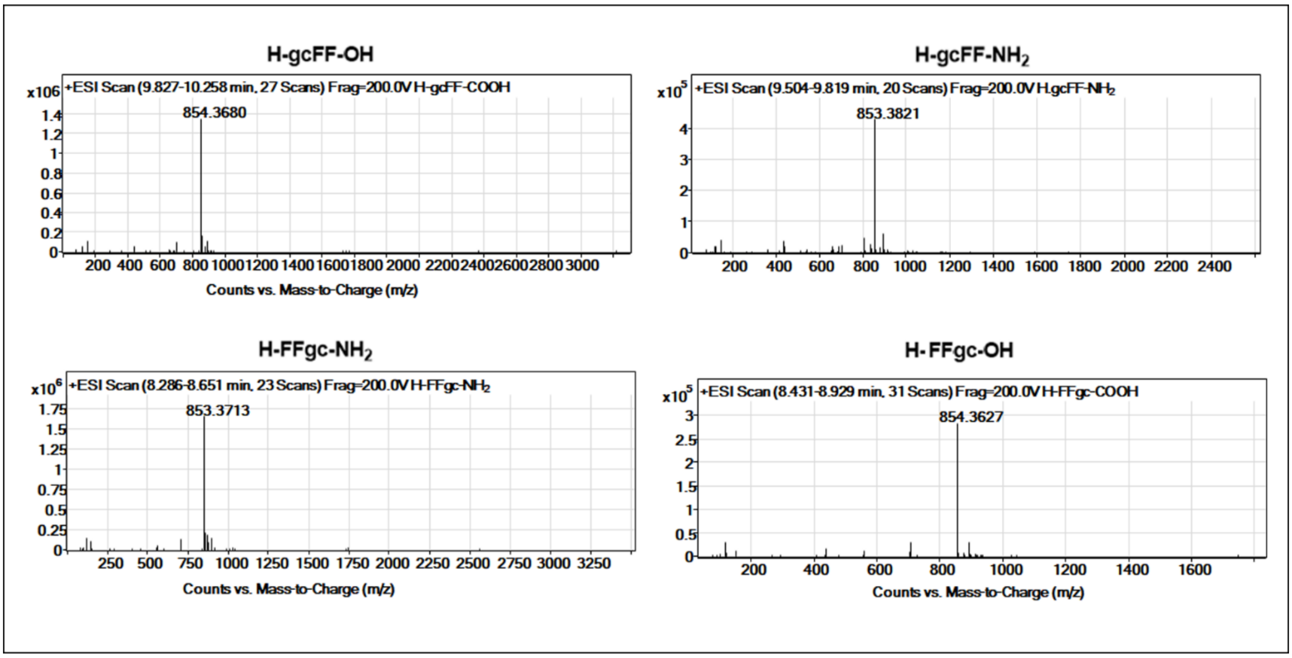

Figure S2: ESI mass spectra for hybrid PNA-peptide derivatives

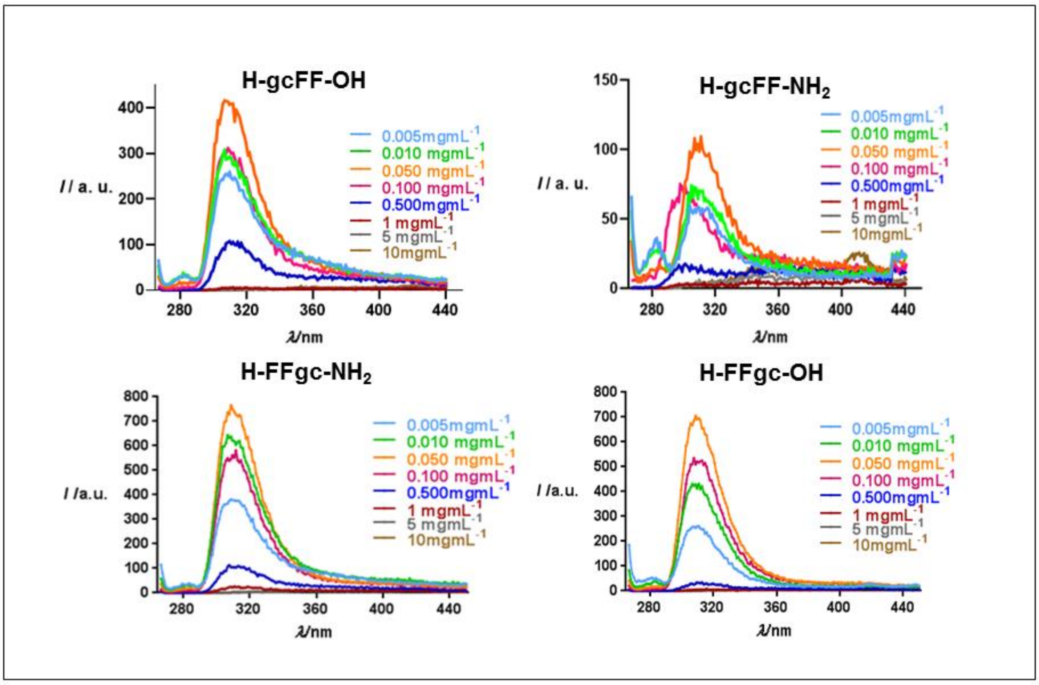

Figure S3: Fluorescence emission spectra of PNA-peptide derivatives excited at λ = 257 nm in 0.005-10.0 mg/mL concentration range.

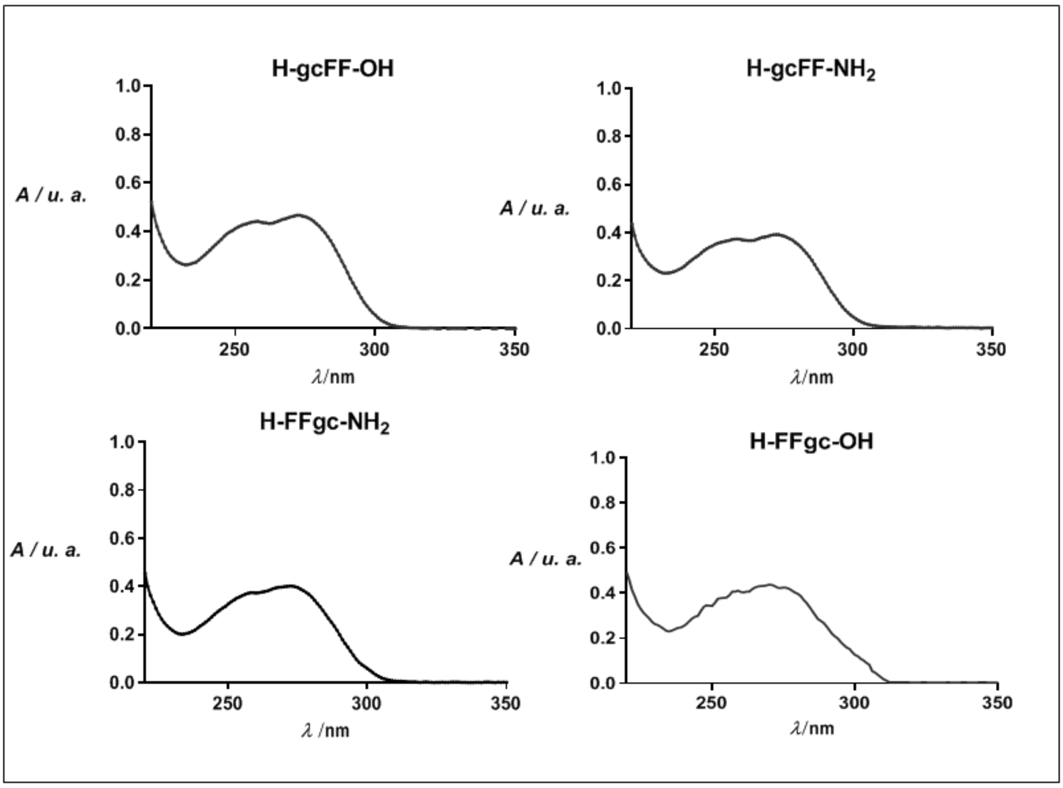

Figure S4: UV-Vis spectra for hybrid PNA-peptide derivatives between 220 an 350 nm.

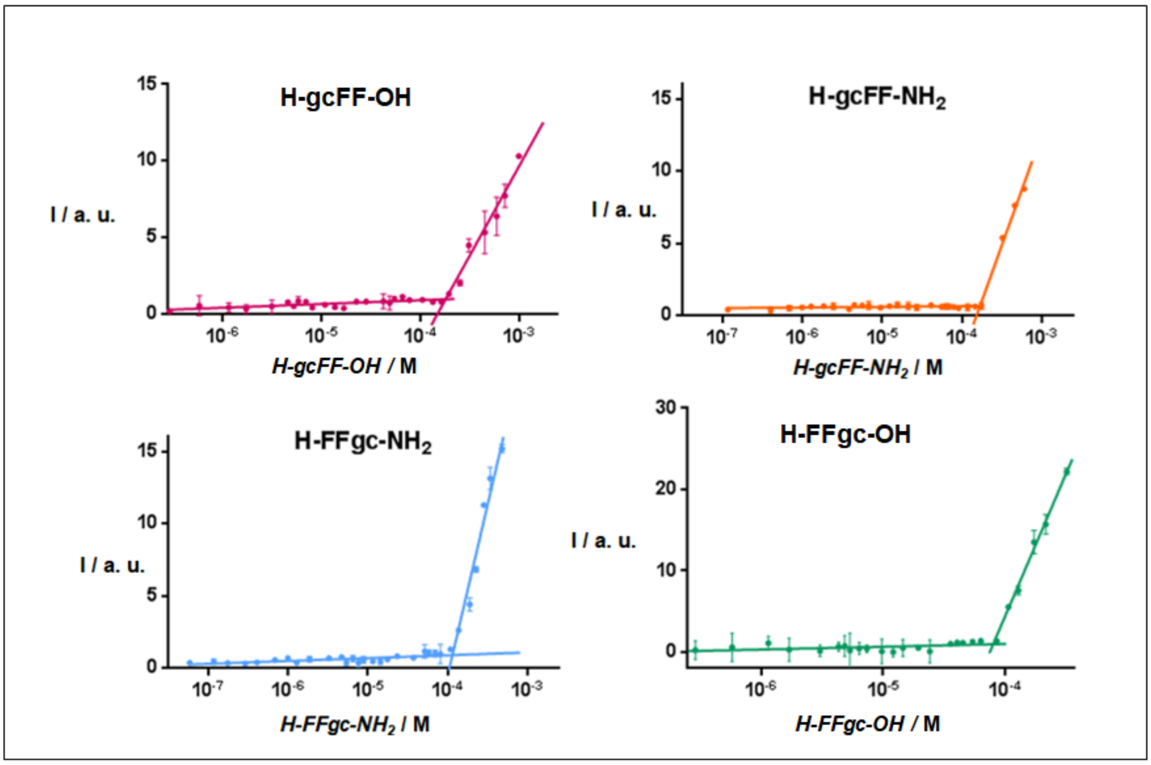

Figure S5: CAC determination for self-assembled aggregates of PNA-peptide derivatives. Plots of the fluorescence intensity emission of the ANS fluorophore in the maximum at 470 nm versus concentration of each peptide.

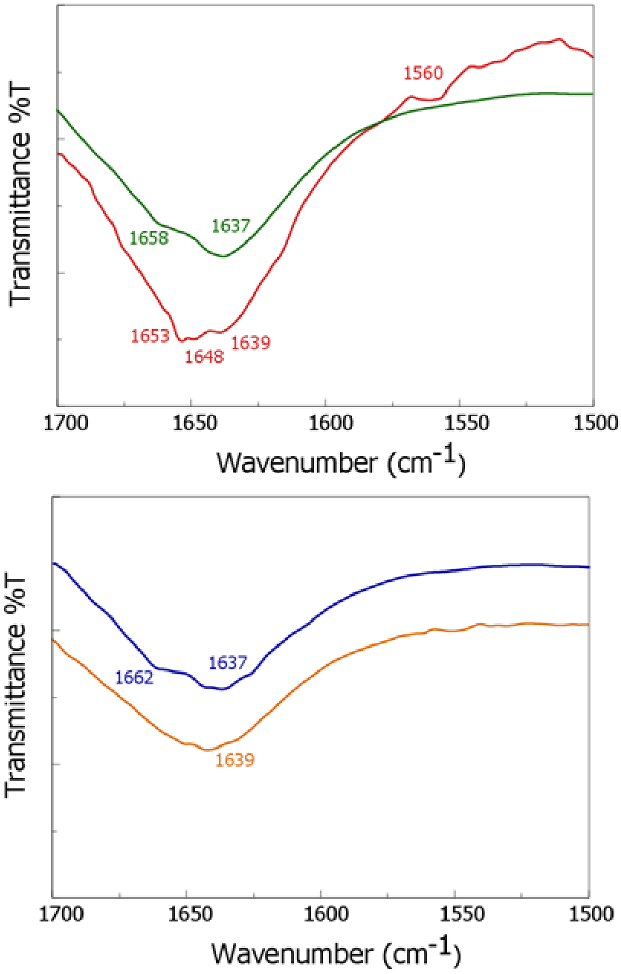

Figure S6: FTIR spectra in the region between 1700-1500 cm^{-1} for PNA-FF conjugates at 5.0 $mg\cdot mL^{-1}$ concentration: H-gcFF-OH (red), H-gcFF-NH_2 (green), H-FFgc-OH (blue) and H-FFgc-NH_2 (orange).

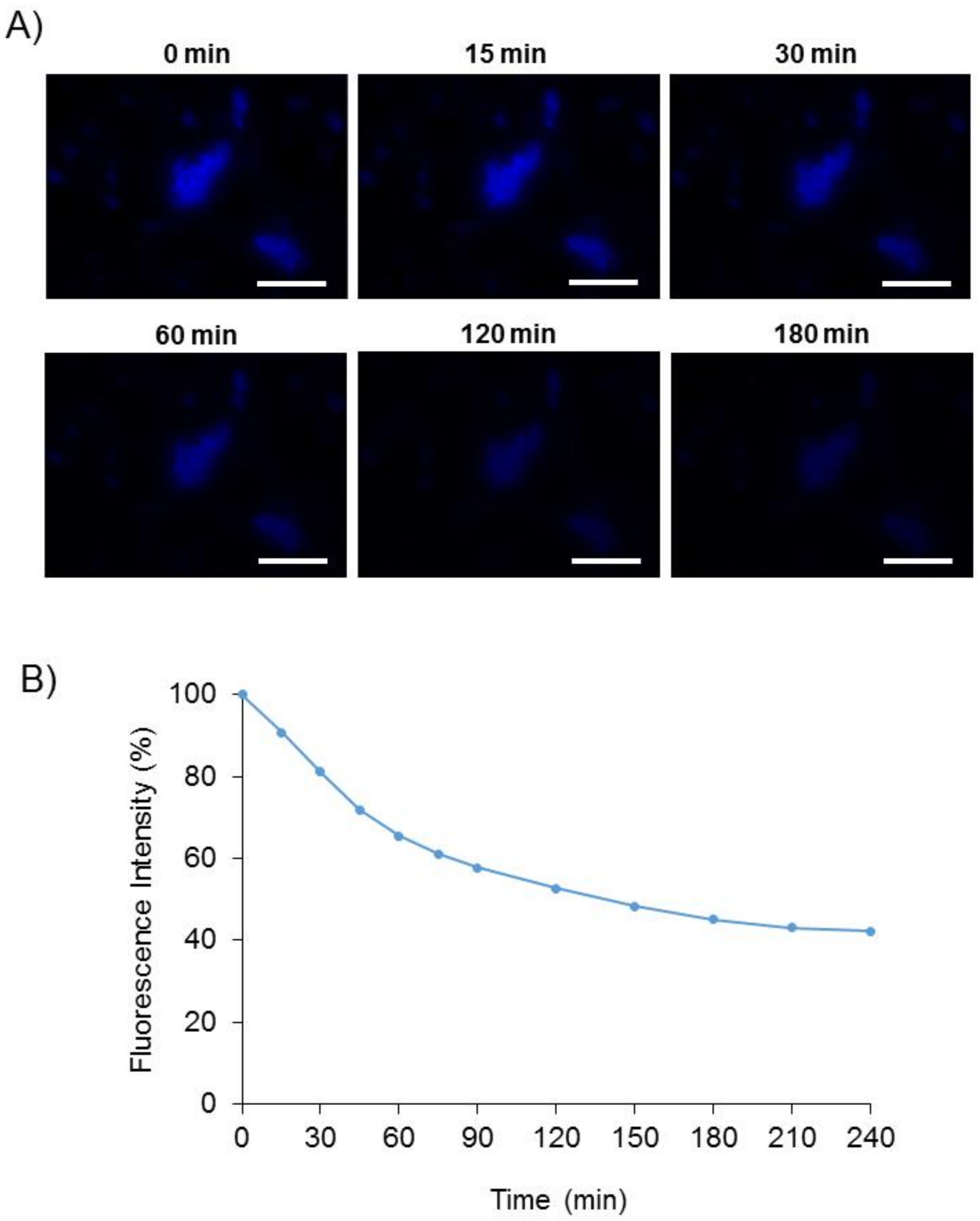

Figure S7: (A) Fluorescence microscopy images of the self-assembled H-gcFF-OH derivative drop-casted on glass slides and air-dried at room temperature. Images are obtained by exciting the sample in the spectral region of DAPI and keeping the light on for 240 min. In the figure are reported fluorescence images at selected time points (0, 15, 30, 60, 120, 180 min). The scale bar of all the images is 50 µm. (B) Percentage decrease of the fluorescence intensity as function of the time of the sample under exposition to continuos light.

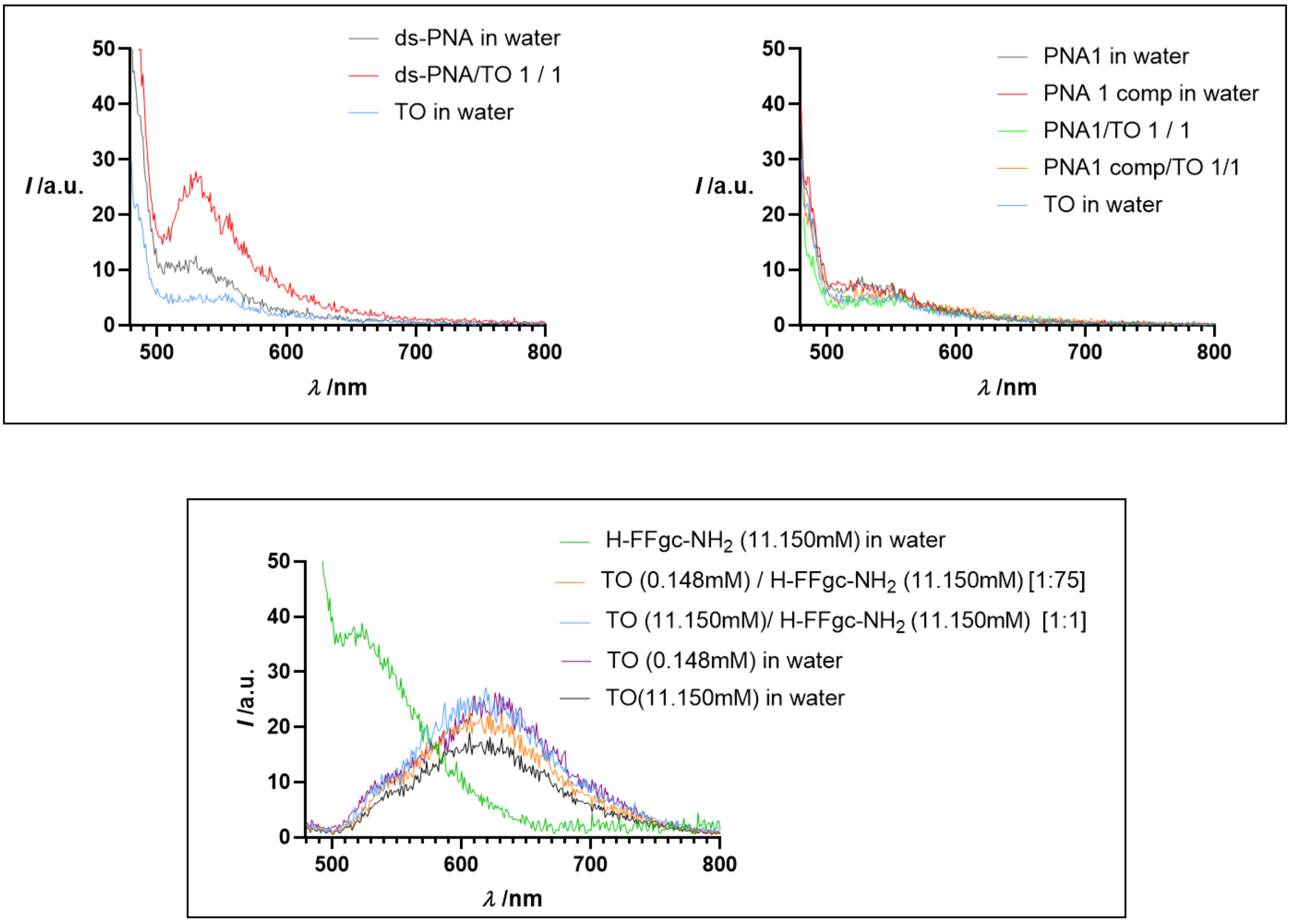

Figure S8: binding of TO to a PNA duplex, ds-PNA and to PNA single strands (PNA 1 and PNA 1 comp) (top) and to H-FFgc-NH_2 assembly (bottom).

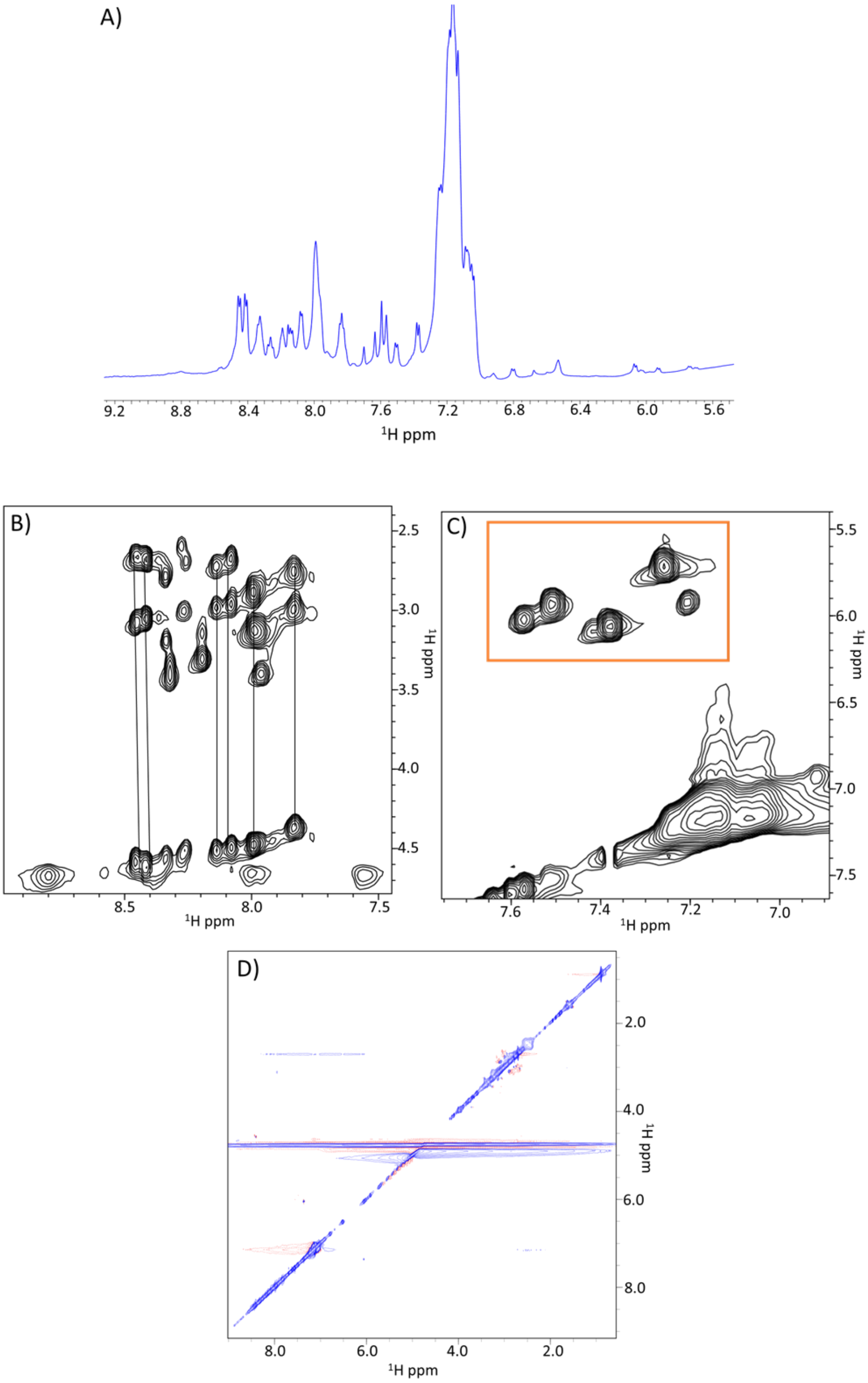

Figure S9: NMR characterization of H-gcFF-OH in H_2O/D_2O 90/10 (2.16 mM concentration). A) 1D [^{1}H] spectrum. B, C) 2D [^{1}H, ^{1}H] TOCSY spectrum; correlations involving H_N and aromatic protons are shown in panels B) and C), respectively. The vertical lines in B) go across different Phe spin systems whereas, the orange rectangle in C) highlights diverse H5-H6 correlations in cytosine. D) 2D [^{1}H, ^{1}H] NOESY 300 spectrum.

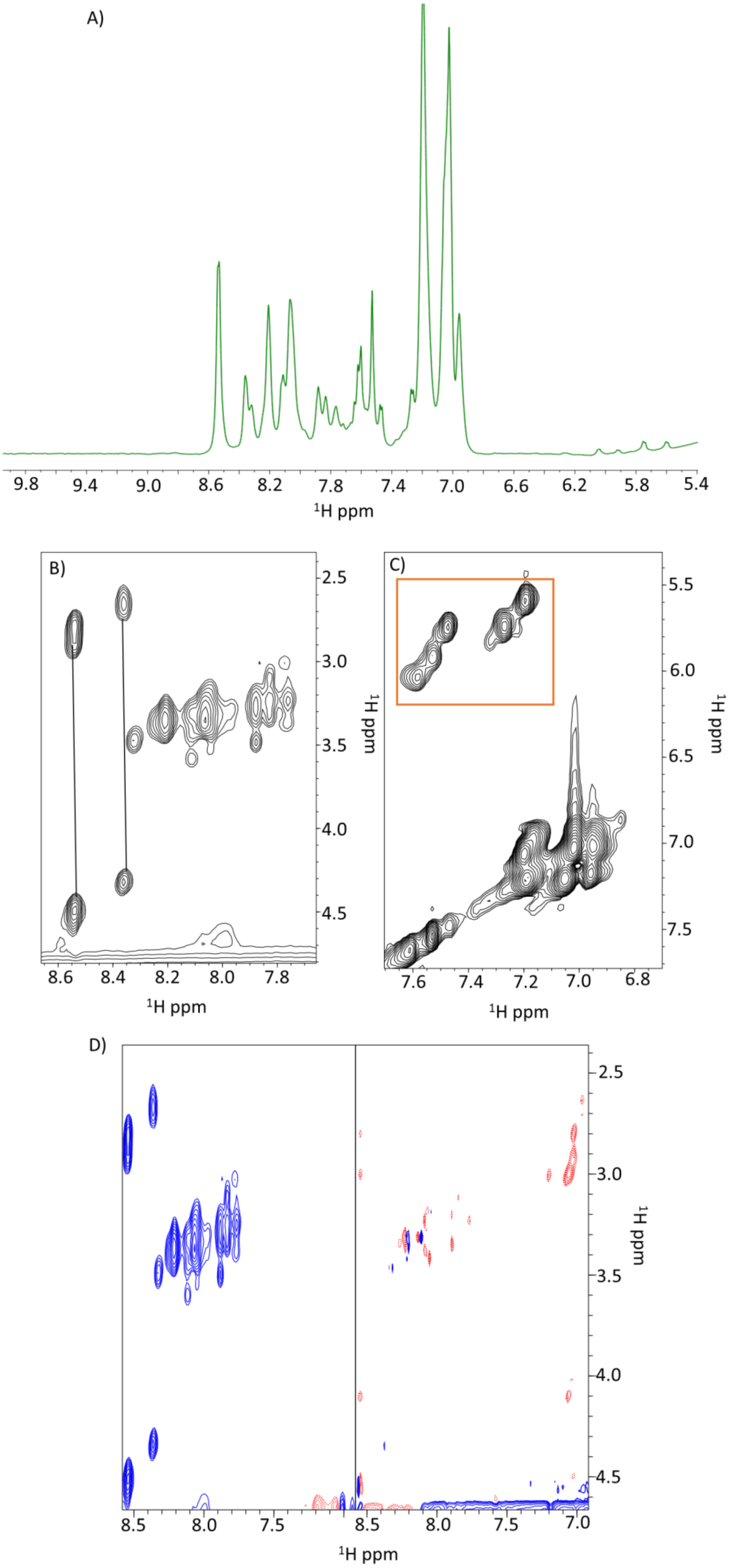

A)
9.8
9.4
9.0
8.6
8.2
7.8
7.4
7.0
6.6
6.2
5.8
5.4
¹H ppm
B)
8.6
8.4
8.2
8.0
7.8
¹H ppm
2.5
3.0
3.5
4.0
4.5
C)
7.6
7.4
7.2
7.0
6.8
¹H ppm
5.5
6.0
6.5
7.0
7.5
¹H ppm
D)
8.5
8.0
7.5
8.5
8.0
7.5
7.0
¹H ppm
2.5
3.0
3.5
4.0
4.5
¹H ppm

Figure S10: NMR characterization of H-FFgc-OH in H_2O/D_2O 90/10 (1.5 mM concentration). A) 1D [^1H] spectrum. B, C) 2D [^1H, ^1H] TOCSY spectrum. Expansions showing correlations from H_N and aromatic protons are reported in panels B) and C), respectively. The vertical lines in B) pass through different Phe spin systems whereas, the orange rectangle in C) highlights diverse H5-H6 correlations in cytosine. D) Comparison of 2D [^1H, ^1H] TOCSY (left side) and NOESY 300 spectrum (right side). Blue peaks are of the same sign of the diagonal peaks, red peaks have opposite sign respect to the diagonal.

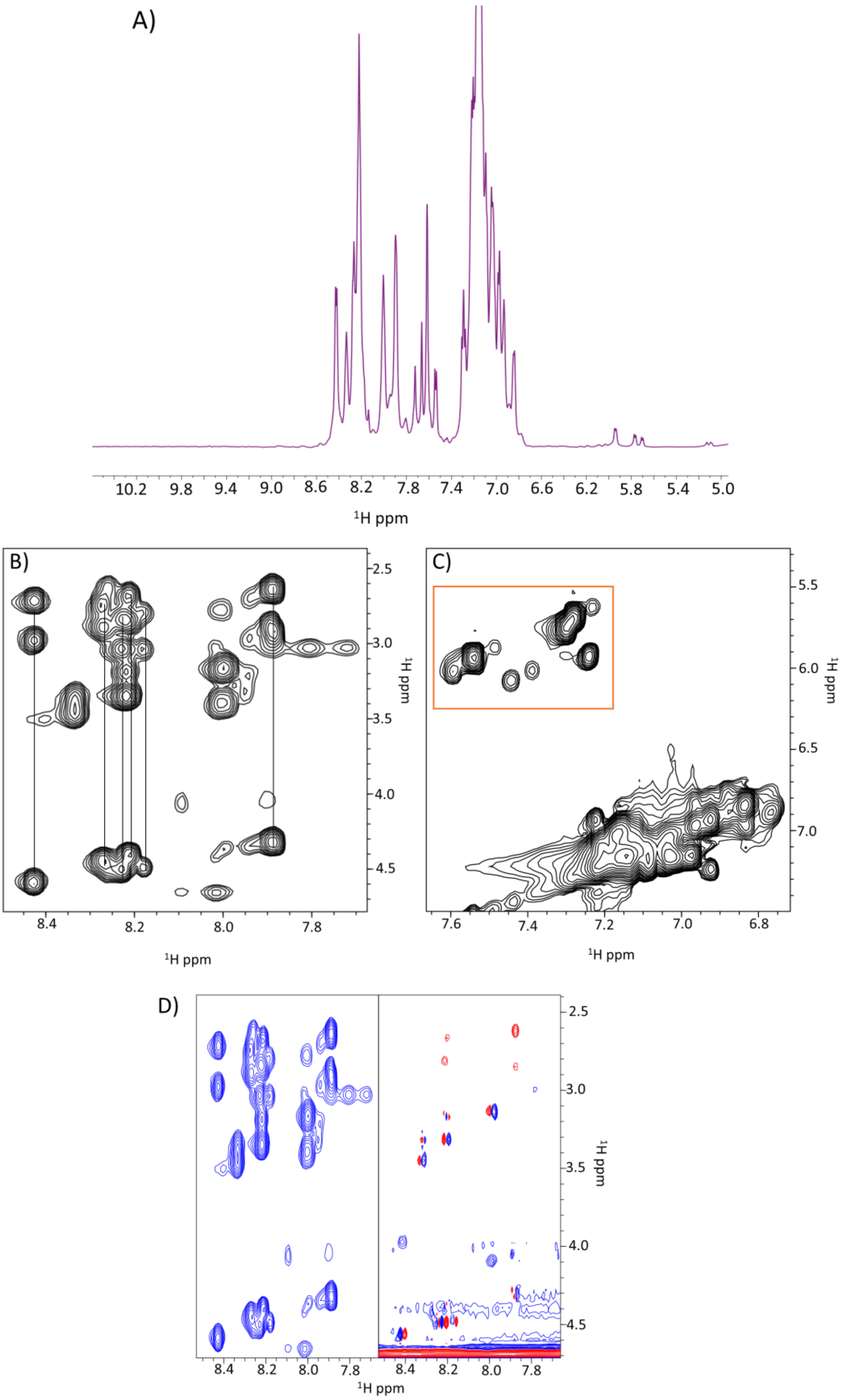

Figure S11: NMR characterization of H-gcFF-NH_2 in H_2O/D_2O 90/10 (1.73 mM concentration). A) 1D [^{1}H] spectrum. B, C) 2D [^{1}H, ^{1}H] TOCSY spectrum: correlations involving H_N and aromatic protons are shown in panels B) and C), respectively. The vertical lines in B) highlight different Phe

spin systems whereas, the orange rectangle in C) includes H5-H6 correlations arising from the aromatic protons in the cytosine. D) Comparison of 2D [^{1}H, ^{1}H] TOCSY (left side) and NOESY 300 spectrum (right side). Blue peaks have the same sign of the diagonal peaks, red peaks have opposite sign respect to the diagonal.

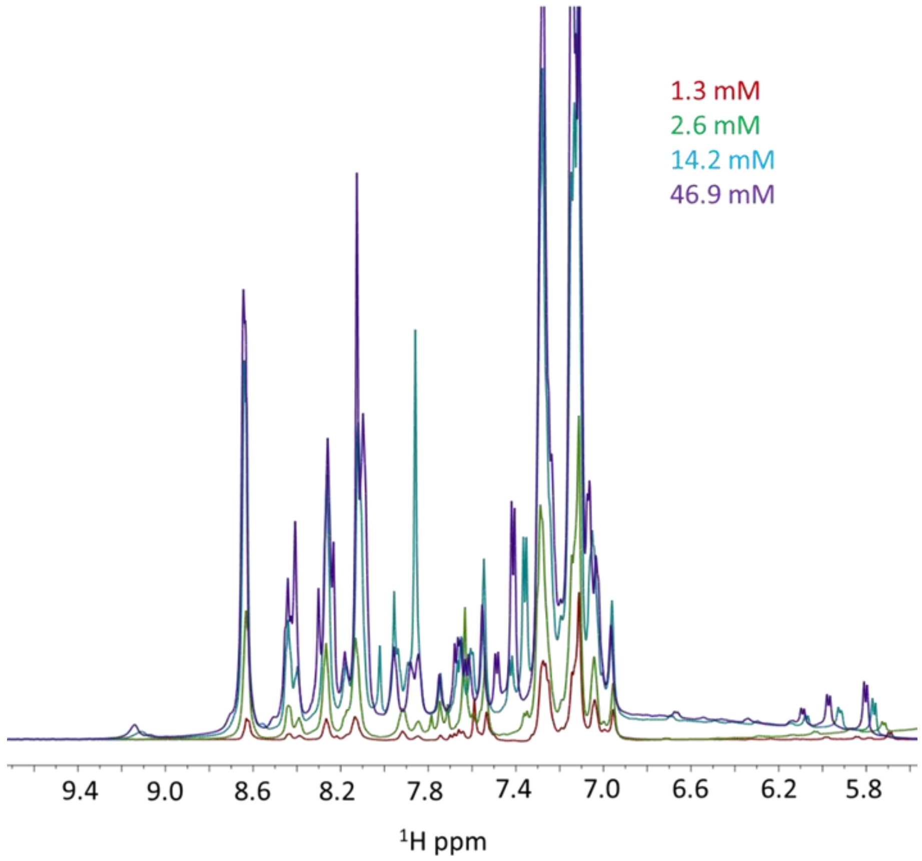

Figure S12: Overlay of 1D [^{1}H] NMR spectra of H-FFgc-NH_2 in H_2O/D_2O 90/10 at different concentrations (i.e., 1.3 (red), 2.6 (green), 14.2 (turquoise), 46.9 (violet) mM). The spectral region containing signals from HN and aromatic protons is shown.

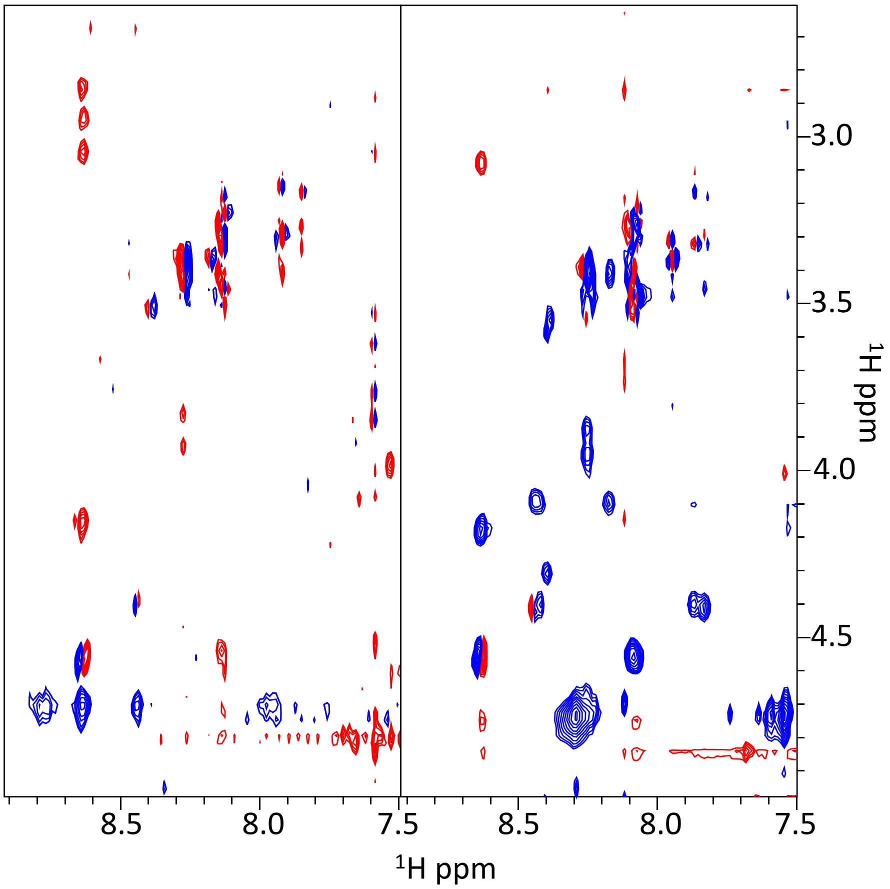

Figure S13: Comparison of NOESY 300 spectra of H-FFgc-NH_2 acquired at 1.3 mM concentration (left) and 46.9 mM concentration (right). Red contours indicate peaks with opposite sign with respect to the diagonal.

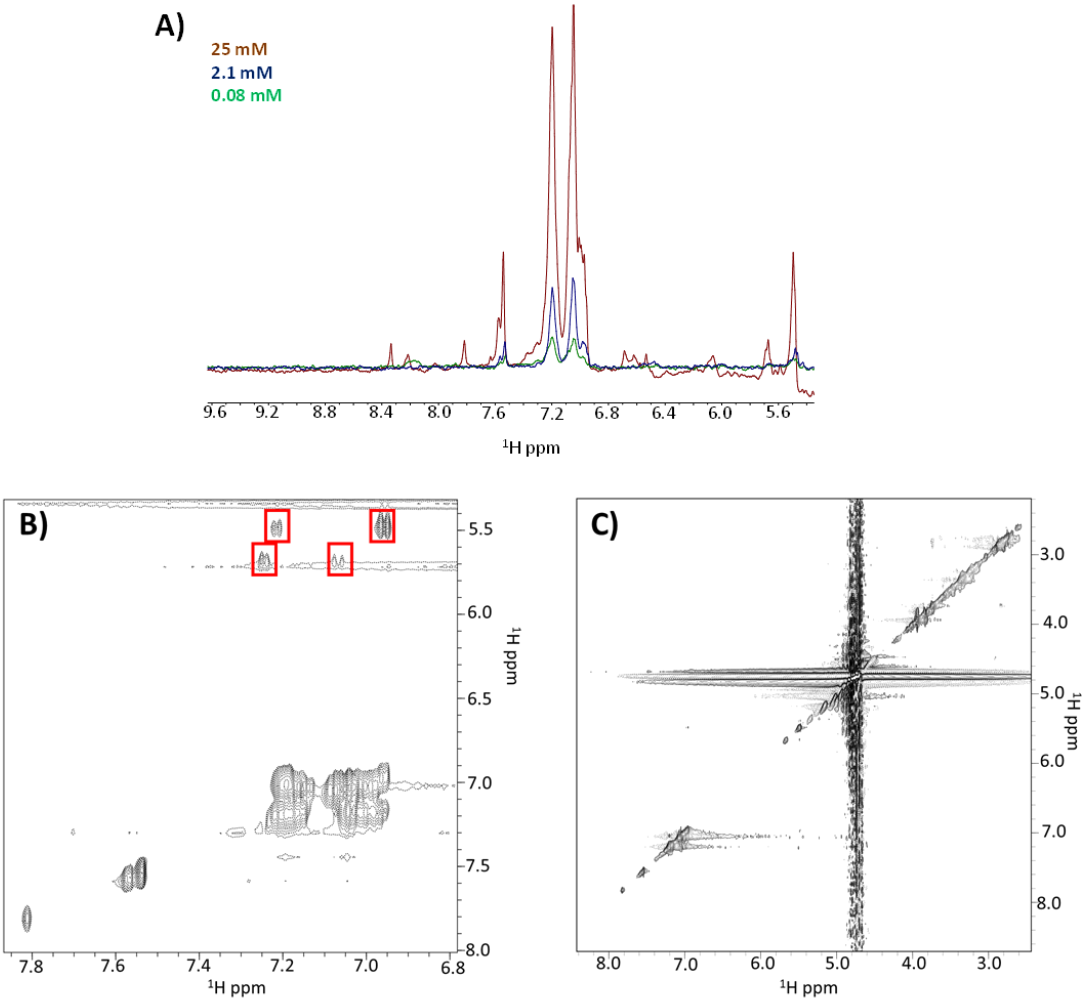

Figure S14: NMR studies in 50 mM sodium phosphate at pH=7.1. A) 1D [^{1}H] NMR spectra of H-FFgc-NH_2 at 25 (red), 2.1 (blue) and 0.08 (green) mM concentration: the spectral region containing aromatic proton signals is shown. B) Expansion of the 2D [^{1}H, ^{1}H] TOCSY spectrum of H-FFgc-NH_2 at 25 mM concentration where H5-H6 correlations in between aromatic protons of citosine in different rotamers have been highlighted with a red box. C) 2D [^{1}H, ^{1}H] NOESY 300 spectrum of H-FFgc-NH_2 at 25 mM concentration.

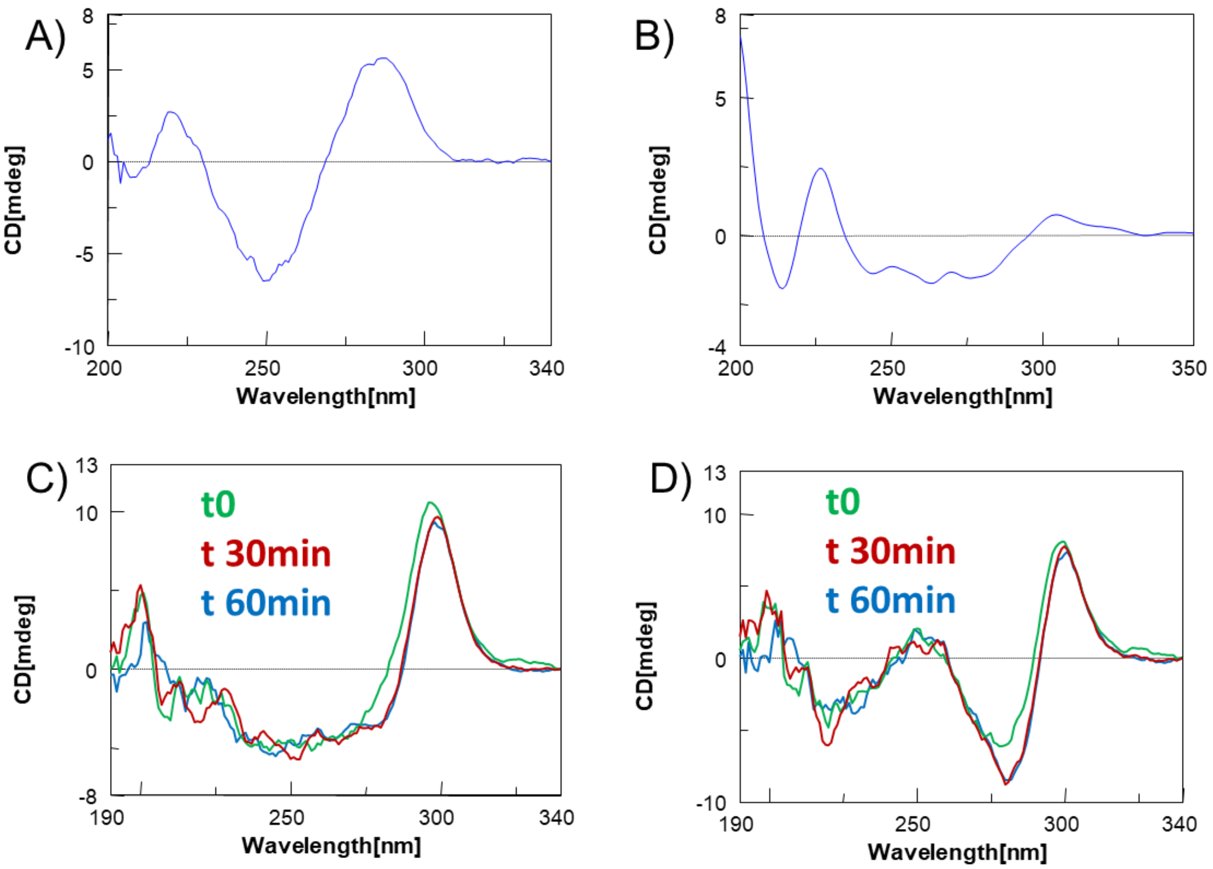

Figure S15: DNA/aggregate binding. CD spectra recorded at 25°C in water of A) ssDNA, B) H-gcFF-OH, C) mixture of ssDNA and H-gcFF-OH (1/10, mol/mol) after annealing at different times, D) spectra obtained subtracting the ssDNA spectrum to those of the annealed mixture composed of ssDNA and H-gcFF-OH.

Table S1. [1]H Chemical shifts (± 0.01 ppm) of the main H-FFgc-NH_2 conformer (46.9 mM concentration in H_2O/D_2O 90/10 v/v). Chemical structure with atom nomenclature is given on top.

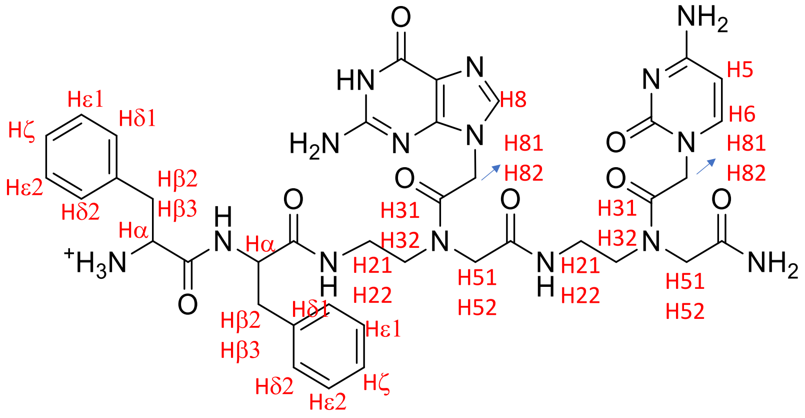

Residue	H_N	Hα	Hβ			Others
**F1**		4.18	3.08<br>3.00			Hδ1 7.14<br>Hδ2 7.14
**F2**	8.64	4.55	2.97<br>2.87			Hδ1 7.11<br>Hδ2 7.11
	**H_N**	**H31-H32<br>H21-H22**		**H51-H52**	**H81-H82**	**Others**
**g3**	8.09	3.46-3.29		3.97-3.87		H8 7.84
**c4**	8.26	3.42-3.50		4.00	4.67	H5 5.79<br>H6 7.41<br>$CONH_2$ 7.55-6.97

References

[1] V. Bezzerri, C. Avitabile, M. C. Dechecchi, I. Lampronti, M. Borgatti, G. Montagner, G. Cabrini, R. Gambari, A. Romanelli, *J. Pept. Sci.* **2014**, *20*, 822–830.

[2] C. Avitabile, L. Moggio, L. D. D'Andrea, C. Pedone, A. Romanelli, *Tetrahedron Lett.* **2010**, *51*, 3716–3718.

[3] E. De Vendittis, G. Palumbo, G. Parlato, V. Bocchini, *Anal. Biochem.* **1981**, *115*, 278–286.

[4] A. Sinopoli, A. Giuffrida, M. F. Tomasello, M. L. Giuffrida, M. Leone, F. Attanasio, F. Caraci, P. De Bona, I. Naletova, M. Saviano, A. Copani, G. Pappalardo, E. Rizzarelli, *ChemBioChem* **2016**, *17*, 1541–1549.
